# Beyond digital investment: How value embedding modes shape ESG performance in energy-intensive firms

**DOI:** 10.1371/journal.pone.0343345

**Published:** 2026-09-21

**Authors:** Weiran Sun

**Affiliations:** School of Management, Nanjing University of Posts and Telecommunications, Nanjing, Jiangsu, China; University of Almeria: Universidad de Almeria, SPAIN

## Abstract

There is no consensus in existing literature on how digital transformation facilitates sustainable development in energy-intensive enterprises. Based on the Resource-Based View and Dynamic Capabilities Theory, this paper proposes the “Value Embedding Mode” analytical framework, which categorizes the value conversion pathways of digital resources into asset-embedded and information-embedded pathways, and empirically examines this framework using panel data from listed companies in China's energy-intensive industries from 2011 to 2024. The study finds a positive association between digital transformation and ESG performance; however, this relationship does not simply stem from the scale of digital investment but depends on whether digital resources can realize value conversion through effective embedding mechanisms. Specifically, the asset-embedded pathway uses green innovation as a mediating channel; however, due to structural factors such as the capital-intensive nature of energy-intensive industries and long asset renewal cycles, its mediating effect is not robustly supported. In contrast, the information-embedded pathway, which relies on improvements in the external information environment as its channel, exhibits more stable effects on value conversion. This paper reveals the underlying logic by which the same digital resources generate differentiated ESG effects depending on the embedding objects and value realization approaches, offering a new theoretical perspective on the heterogeneous outcomes of digital transformation in energy-intensive enterprises.

## Introduction

Against the backdrop of China's green transition, "promoting green and low-carbon economic and social development has become a key requirement for achieving high-quality development" [1]. This policy orientation highlights the central role of green development in China's pursuit of modernization and has elevated the "dual carbon" goals into a comprehensive transformation of the economic and social system. In this transformation process, energy-intensive industries, characterized by substantial energy consumption and carbon emissions, have become focal sectors of China's green transition initiatives and representative contexts for assessing transition effectiveness [2]. Relevant data indicate that energy-intensive industries have long accounted for approximately 30% of total industrial output value, while their carbon emissions exceed 80% of industrial emissions. Given China's energy endowment characterized by "abundant coal, scarce oil, and limited natural gas," these industries have maintained a high dependence on fossil fuels over an extended period [3–5]. Therefore, energy-intensive firms are not only critical for achieving the "dual carbon" goals but also provide a representative research setting for examining how digital transformation contributes to corporate ESG performance. However, compared with general firms, energy-intensive enterprises exhibit distinctive characteristics, including capital intensity, high asset specificity, and long equipment replacement cycles. Consequently, whether digital resources can be effectively transformed into ESG value is deeply constrained by industry-specific structural conditions. Therefore, it remains unclear whether digital transformation can consistently enhance ESG performance in energy-intensive firms and whether different value realization mechanisms are subject to equivalent structural constraints.

Digital transformation is viewed as a key pathway to overcoming the challenges of corporate green transition, but there remains some uncertainty regarding the practical effectiveness of the "digital investment—ESG improvement" approach for energy-intensive enterprises. On the one hand, the empowerment perspective argues that digital technologies can provide new drivers for improving corporate ESG performance by optimizing business processes, enhancing the efficiency of resource allocation, and strengthening organizational capabilities [6,7]. At the same time, by increasing risk-taking capacity, optimizing human capital, and alleviating financing constraints, digital technologies systematically reshape enterprises' capabilities for green innovation and sustainable development, thereby forming a technology-driven pathway of "digitalization—green innovation—ESG improvement" [8–12]. On the other hand, the cost-constraint perspective points out that digital transformation requires sustained, substantial investment in infrastructure construction, technology system development, and organizational restructuring. However, energy-intensive enterprises already face significant green governance pressures and resource constraints [13,14]. Investments in digital transformation may alter a company's resource allocation structure and, in the short term, create competition for green governance resources, thereby undermining the sustainable value of digital transformation [5,15]. Furthermore, the context-dependent perspective further notes that the relationship between digital transformation and corporate performance is not a simple linear one but may be influenced by factors such as a firm's ability to integrate resources and its organizational adaptability [16–18]. This suggests that the impact of digital transformation on ESG performance is not simply determined by technological investment; its effects may depend on the conditions under which different transmission mechanisms are realized in specific industry contexts [19].

Upon further analysis, it becomes clear that this theoretical divergence is not coincidental; it reflects the fact that the mechanisms through which digital transformation influences ESG performance have not yet been fully elucidated. Although existing research has explained the economic and environmental effects of digital transformation from perspectives such as efficiency gains, capability restructuring, and cost constraints, there remains a lack of systematic comparison regarding the effectiveness of different transmission mechanisms in specific industry contexts. In particular, existing research has focused more on how digitalization drives corporate sustainability through technological pathways such as green innovation, while paying insufficient attention to the governance value inherent in digitalization and its underlying mechanisms.

In fact, the value of digital transformation extends beyond technological innovation. In recent years, the development of digital infrastructure, artificial intelligence, and the Industrial Internet has gradually expanded how digital transformation creates value from "efficiency gains" to "governance restructuring." An increasing number of studies are beginning to focus on the institutional value of digital technologies in enhancing corporate transparency, improving stakeholder communication, and strengthening external oversight—rather than being limited solely to improvements in production efficiency. However, there remains a lack of systematic discussion regarding the mechanisms through which this governance value operates in energy-intensive industries. For energy-intensive enterprises, the environmental governance process is highly specialized, complex, and opaque; external stakeholders often find it difficult to accurately assess a company's actual fulfillment of environmental responsibilities, resulting in significant information asymmetry. Digital transformation can make corporate environmental behavior more observable and evaluable by enhancing data processing capabilities, improving the efficiency of information transmission, and optimizing the external information environment. This reduces the cost for stakeholders to access ESG-related information and strengthens external oversight and evaluation mechanisms. Therefore, the external information environment may constitute an important transmission channel through which digital transformation influences ESG performance. It should be clarified in advance that this paper uses the concept of the external information environment to characterize this governance transmission channel, referring to the accessibility and analyzability of a firm's ESG-related information to external parties, rather than the quality of the firm's information disclosure itself; the correspondence between its operational measurement and theoretical implications will be addressed in Chapter 4.

Looking further, structural constraints in energy-intensive industries may lead technological and governance-oriented pathways to exhibit differentiated value realization effects. The green innovation pathway relies on long-term technological accumulation, capital investment, and innovation resource allocation. It requires the further transformation of digital resources into technological outcomes and their integration into corporate production systems. In contrast, the information governance pathway relies more on firms' information processing capabilities and external communication efficiency, realizing value creation by improving processes of information generation, transmission, and governance. Because these two pathways rely on different underlying conditions, their value realization processes may be affected by distinct constraints in energy-intensive industries.

For energy-intensive firms, the further embedding of digital resources into production systems is often constrained by industry-specific structural characteristics. Capital intensity, high asset specificity, and long equipment replacement cycles increase the adjustment costs associated with corporate physical assets. This study conceptualizes these structural constraints as "asset rigidity," referring to resource conversion constraints arising from capital commitment, asset specificity, and lengthy replacement cycles. Specifically, asset rigidity is reflected in potential barriers to transforming digital resources into green innovation outcomes. For example, in capital-intensive industries such as steel and cement, production equipment typically has long replacement cycles, and green technology upgrading requires sustained and substantial capital investment. As a result, the transformation of digital resources into green innovation outcomes may be hindered by asset lock-in effects. Meanwhile, in technology-intensive industries such as chemicals and non-ferrous metals, although the green transition requires substantial technological capabilities, green technology resources are also highly concentrated: the top 20% of firms in these sectors control more than 60% of core green technology resources [20]. Limited by insufficient technological accumulation and innovation resources, small and medium-sized enterprises (SMEs) may find it difficult to rapidly develop competitive advantages in green innovation through digital investment alone. Therefore, although different segments within energy-intensive industries face distinct constraints, these structural characteristics collectively increase the difficulty of transforming digital resources into green innovation outcomes.

It is worth noting that asset rigidity does not mean that digital transformation cannot create ESG value; rather, it affects the efficiency of value realization across different pathways. The green innovation pathway, which relies on adjustments to the production system—such as equipment upgrades, process optimization, and production workflow reforms—is more susceptible to the constraints of asset rigidity. In contrast, the external information environment pathway relies more on a company's information processing capabilities, the efficiency of information transmission, and the optimization of external communication mechanisms; as such, the realization of its value is relatively less affected by the constraints of the industry's asset structure. Furthermore, the difference between the two is not one of degree but of conversion logic: the value of the former must be realized through the transformation of physical assets [21], while the latter is realized with relatively limited dependence on physical asset adjustments—this distinction determines that industry asset rigidity will impose asymmetric constraints on the two pathways, and it is precisely the starting point for all theoretical deductions and empirical designs in this paper.

Based on the above analysis, this paper focuses on a highly distinctive core proposition: In energy-intensive industries characterized by significant asset rigidity, through which channels is the ESG value of digital resources actually realized? Centering on this proposition, this paper poses three progressive questions: First, does the positive correlation between digital transformation and the ESG performance of high-energy-consumption enterprises exist as a stable direct effect, or is it primarily realized indirectly through transmission mechanisms? Second, can technology-enabled pathways—represented by green innovation—be viable under the structural constraints of capital-intensive, high-energy-consumption industries with long asset renewal cycles? Third, can governance-enabled pathways—represented by the external information environment—circumvent the constraints of physical assets and become the primary channel for unlocking digital ESG value—and why do these two types of pathways exhibit systemic divergence?

To address these questions, this paper adopts a research design comprising "bibliometric analysis—theoretical model construction—empirical testing." First, using the CiteSpace tool, we conduct a knowledge map analysis of relevant domestic and international studies to identify research hotspots and the knowledge structure in the fields of digital transformation and corporate sustainability; Second, using a sample of listed companies in high-energy-consumption industries on China's Shanghai and Shenzhen A-share markets from 2011 to 2024, we construct a dual-pathway analytical framework examining the impact of digital transformation on ESG performance. We introduce green innovation and the external information environment as mediating variables and, through benchmark regression, mediation effect tests, and multiple robustness tests are conducted to systematically examine the relative effectiveness of the two pathways within a unified empirical framework.

## Literature review and identification of research gaps

### Data sources and bibliometric methods

To systematically clarify the knowledge structure and thematic evolution of research on the relationship between digital transformation and corporate ESG performance, this study employs a bibliometric approach to review relevant domestic and international literature. CiteSpace 6.4.R1 software is used to conduct keyword co-occurrence analysis, cluster analysis, and burst term detection. Given that existing studies specifically examining the intersection of digital transformation and ESG performance in China's energy-intensive enterprises remain relatively limited, directly restricting the research scope to this context may hinder a comprehensive understanding of the knowledge structure in this field. Therefore, this study first analyzes the broader research domain concerning digital transformation and ESG, and subsequently identifies research gaps within the energy-intensive context, thereby providing a literature foundation for the development of the theoretical framework and research questions.

The research data are primarily obtained from the China National Knowledge Infrastructure (CNKI) and the Web of Science Core Collection database. For Chinese literature retrieval, a two-stage retrieval strategy was adopted, consisting of an initial topic search followed by secondary screening within the retrieved results. Specifically, "digital transformation" was used as the initial search term, followed by secondary screening using "ESG" as the topic term, with the literature source restricted to journals indexed in the Peking University Core Journal List and CSSCI database. For English-language literature retrieval, search strategies were constructed around terms such as "digital transformation," "ESG," and "sustainability," with the scope limited to SCI/SSCI-indexed journal articles in the Web of Science Core Collection. After removing duplicates and excluding non-academic publications, 233 valid Chinese articles published between 2005 and 2025 and 409 valid English articles published between 2006 and 2025 were identified, resulting in a total sample of 632 core articles. The entire literature retrieval process was completed in December 2025.

### Evolution of research trends: Deepening research driven by global governance

**Fig 1:**
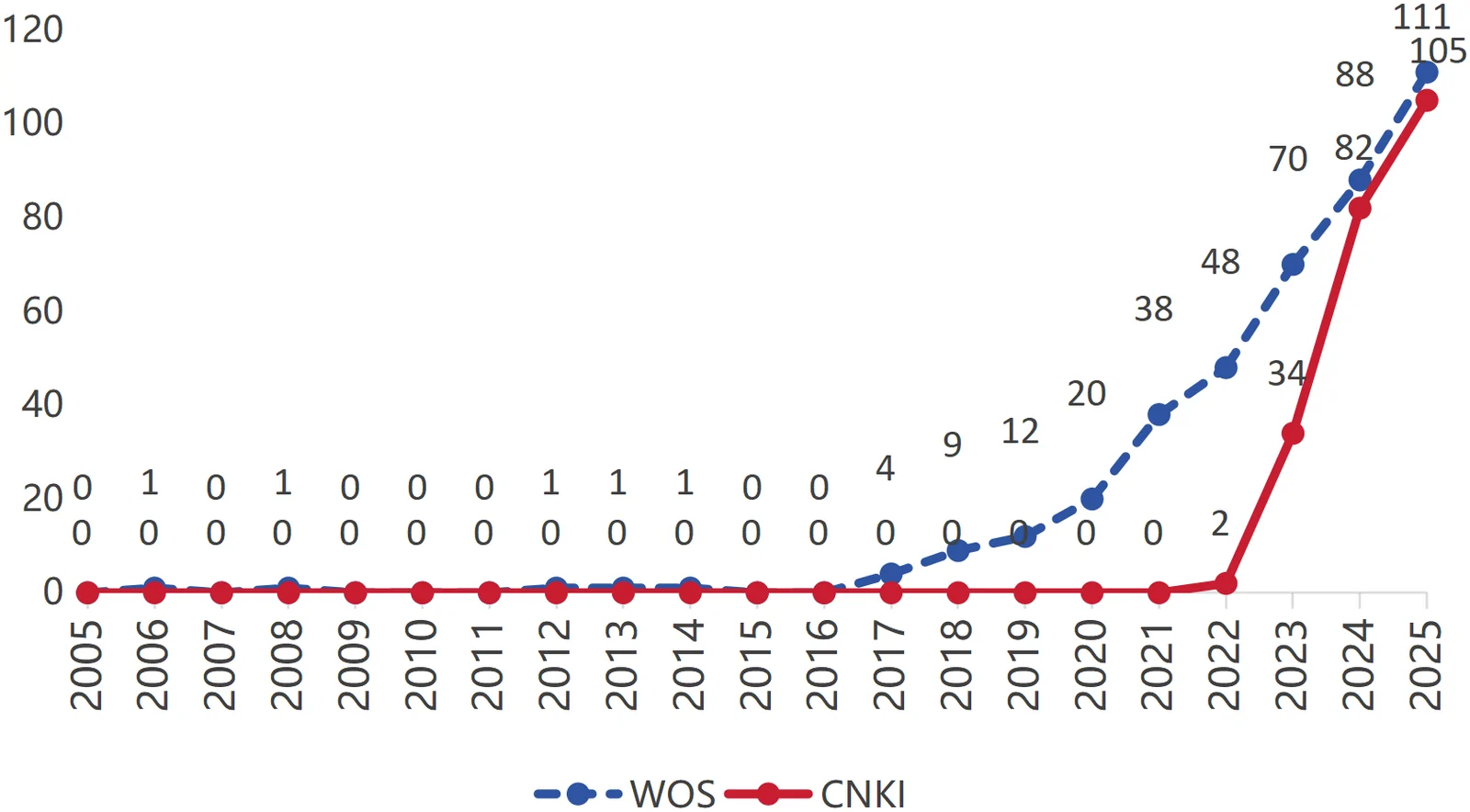
Annual publication volume comparison chart for chinese and English literature, 2005–2025.

Based on trends in the annual publication volume of Chinese and English-language literature from 2005 to 2025 (Fig 1), interdisciplinary research on digital transformation and corporate ESG performance has shown an overall trend of sustained growth, characterized by a gradual evolution from conceptual exploration to a deeper understanding of underlying mechanisms.

From 2005 to 2020, the field was generally in an exploratory phase, with research primarily focusing on the application of digital technologies, corporate innovation capabilities, and sustainable development concepts. In particular, the signing of the Paris Agreement in 2015 propelled global climate governance from target-based initiatives toward institutionalized action, prompting countries to continuously strengthen their carbon emission reduction targets, environmental disclosure requirements, and the development of sustainable development evaluation systems [22,23]. However, research during this phase focused more on the application value of digital technologies and the integration of sustainable development concepts, while systematic analysis of the interactive relationship between the two remained in the preliminary exploratory stage.

Since 2020, with the acceleration of the global green and low-carbon transition and the introduction of China's "Dual Carbon" strategic goals, research on digital transformation and ESG has entered a phase of rapid development. As shown in Fig 1, the volume of Chinese-language literature has exhibited a marked upward trend since 2021, indicating that domestic research in this area is driven by both policy direction and the demands of industrial transformation; at the same time, English-language research has maintained steady growth, with related topics gradually expanding from the application of individual technologies to deeper-level issues such as enhancing innovation capabilities and restructuring organizational capacities.

Overall, research on digital transformation and ESG has gradually shifted from examining the effectiveness of technological applications to identifying underlying mechanisms and boundary conditions. However, a systematic comparison of how different transmission mechanisms vary across specific industry contexts is still lacking. Against this backdrop, energy-intensive enterprises—due to their distinctive asset rigidity—have emerged as a critical context for testing pathways to realizing the value of digital ESG.

### Analysis of knowledge structure

#### Keyword co-occurrence analysis.

**Fig 2:**
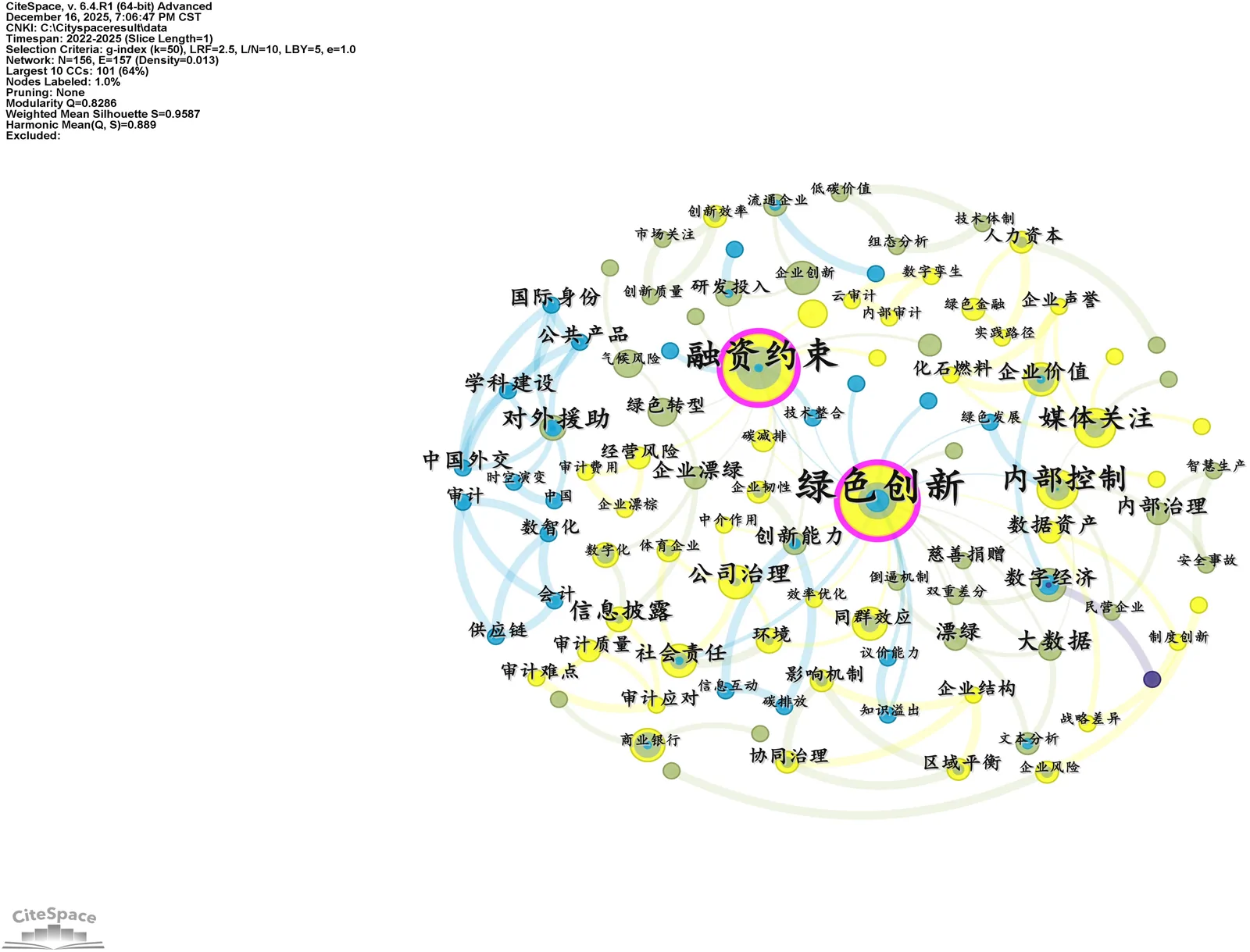
CNKI keyword co-occurrence map.

A keyword co-occurrence network for Chinese literature was constructed based on the CNKI database (Fig 2). The network comprises 156 nodes and 157 links, with a network density of 0.013. The largest connected subgraph contains 101 nodes, accounting for 64% of the entire network, indicating that domestic research has formed a relatively clear structure of thematic interconnections. Keywords such as “green innovation,” “financing constraints,” “digital economy,” and “corporate innovation” occupy key positions in the network. Among these, “green innovation” embodies the technical logic behind the transformation of digital resources into green outcomes, while “financing constraints” reflect a critical mechanism through which digital transformation impacts enterprises’ ability to allocate resources. Meanwhile, the presence of keywords such as “media attention,” “corporate governance,” “information disclosure,” and “social responsibility” indicates that some studies have begun to focus on the governance effects of digital transformation; however, related research still primarily relies on traditional governance perspectives.

**Fig 3:**
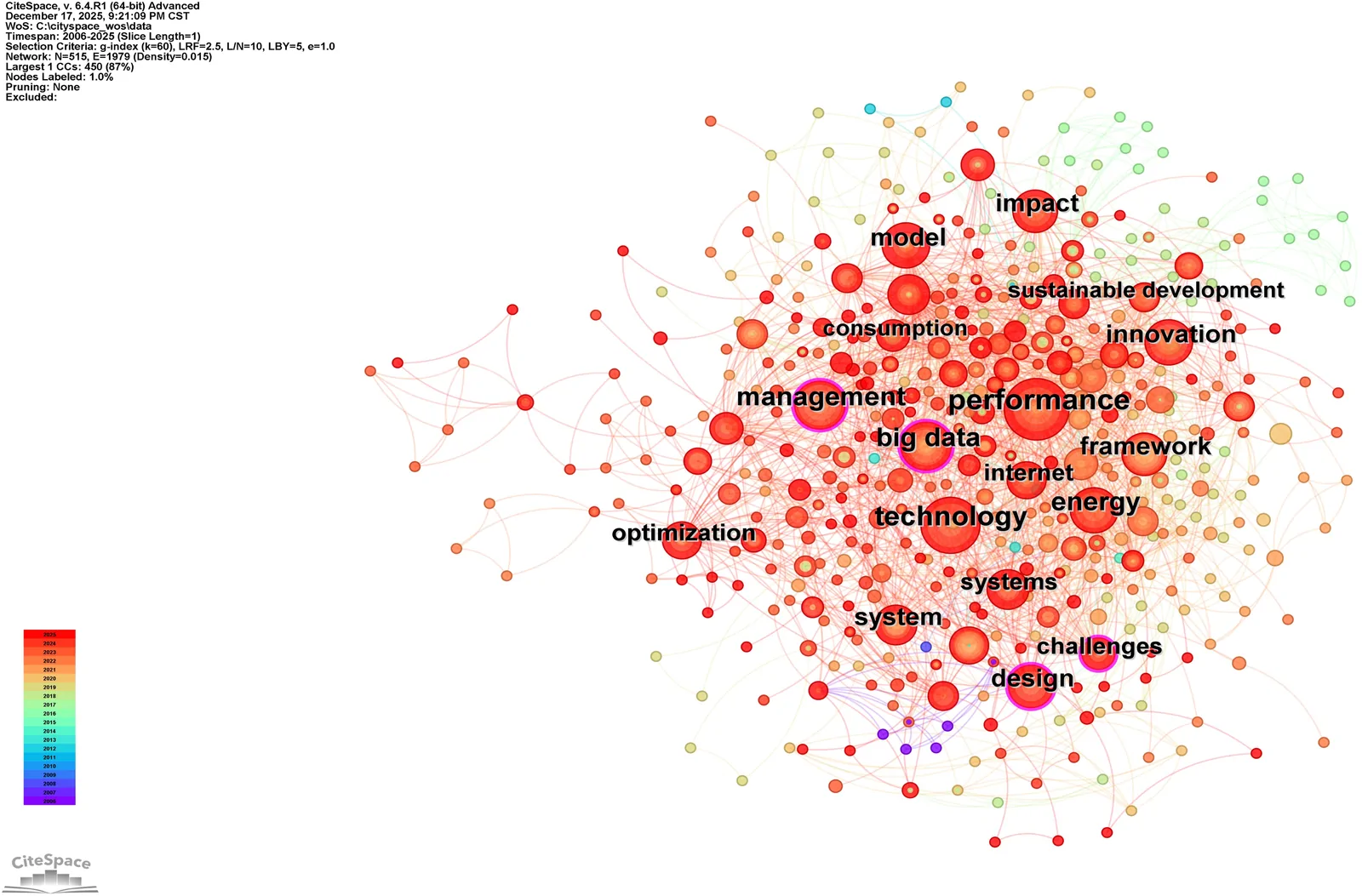
WOS keyword co-occurrence map.

A co-occurrence network of keywords from English-language literature was constructed based on the Web of Science Core Collection (Fig 3). This network comprises 515 nodes and 1,979 links, with a network density of 0.015. The largest connected subgraph contains 450 nodes, accounting for 87% of the total, indicating that the international research network exhibits stronger interconnectivity and thematic expansiveness. Keywords such as “performance,” “technology,” “innovation,” and “sustainable development” occupy the core of the network, while digital technology themes such as “big data” and “the Internet” are closely associated with them. This suggests that international research also focuses on the application of digital technologies, the enhancement of innovation capabilities, and the improvement of sustainable performance. Although governance themes such as “corporate social responsibility” and “information disclosure” are touched upon, systematic research centered on the governance functions of digital information has not yet emerged.

Overall, existing research hotspots are concentrated on the application of digital technologies, the enhancement of innovation capabilities, and the improvement of corporate sustainable performance. Although governance-related themes are gradually attracting attention, they have not yet become a core research direction in this field.

#### Cluster analysis.

**Fig 4:**
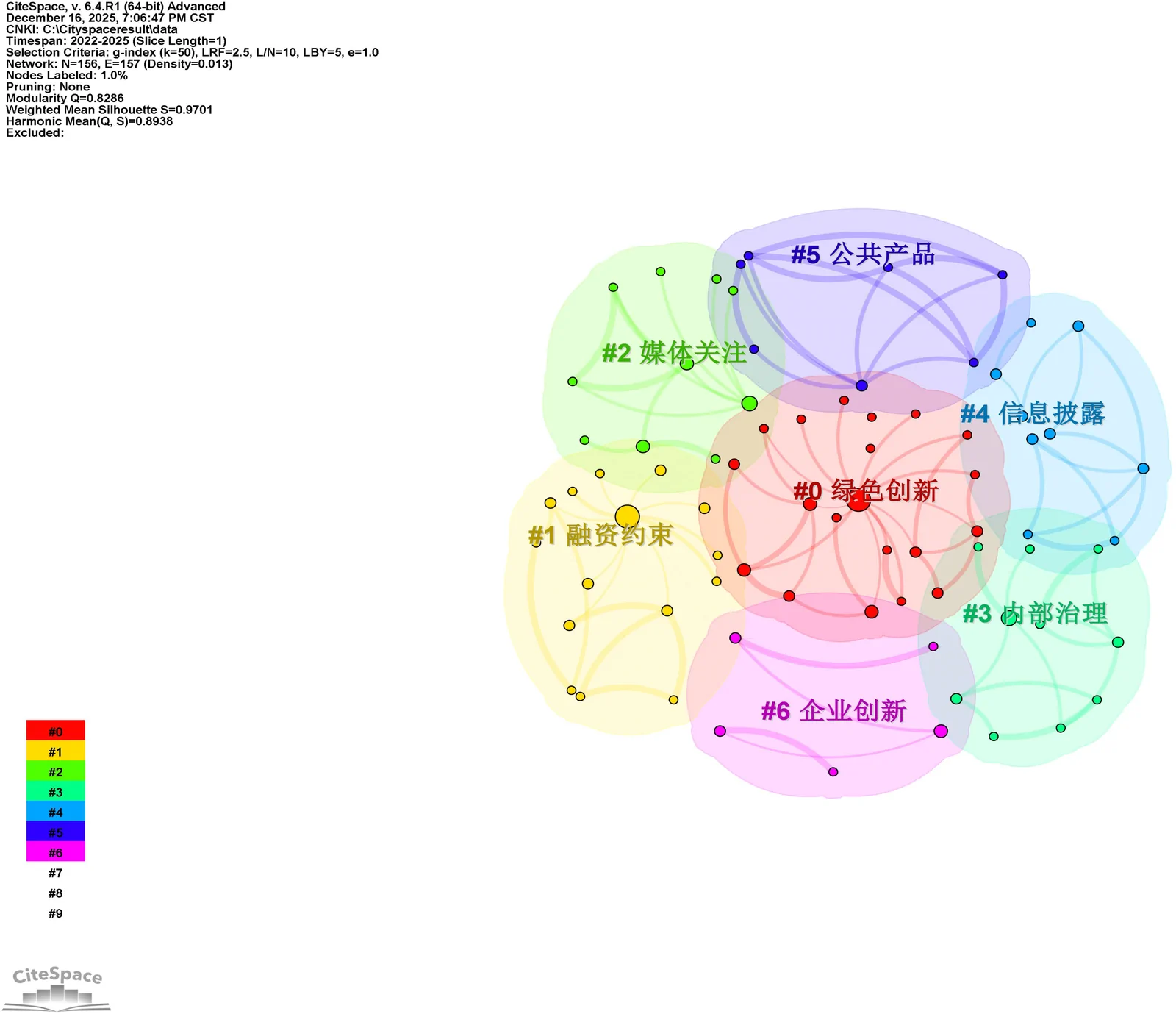
CNKI Chinese literature keyword clustering map.

Building on the co-occurrence analysis, cluster analysis was further conducted to identify the thematic structure of this field. The clustering results for Chinese literature (Fig 4) show a network modularity Q = 0.8286 and a weighted average silhouette coefficient S = 0.9701, indicating that the cluster structure possesses good stability and internal consistency. The analysis identified thematic clusters primarily centered on green innovation, financing constraints, media attention, internal governance, information disclosure, and corporate innovation. In terms of thematic relationships, domestic research has established a value realization logic centered on technological innovation, extending to mechanisms such as resource allocation and corporate governance. Among these, green innovation and corporate innovation reflect the transformation of digital resources into technological achievements; financing constraints reflect the impact of resource acquisition and allocation; and information disclosure and internal governance pertain to governance effects. However, there remains a lack of effective linkage between the themes of technological innovation and governance mechanisms, and the ways in which digitalization achieves ESG value enhancement through information mechanisms have not yet been fully explained.

**Fig 5:**
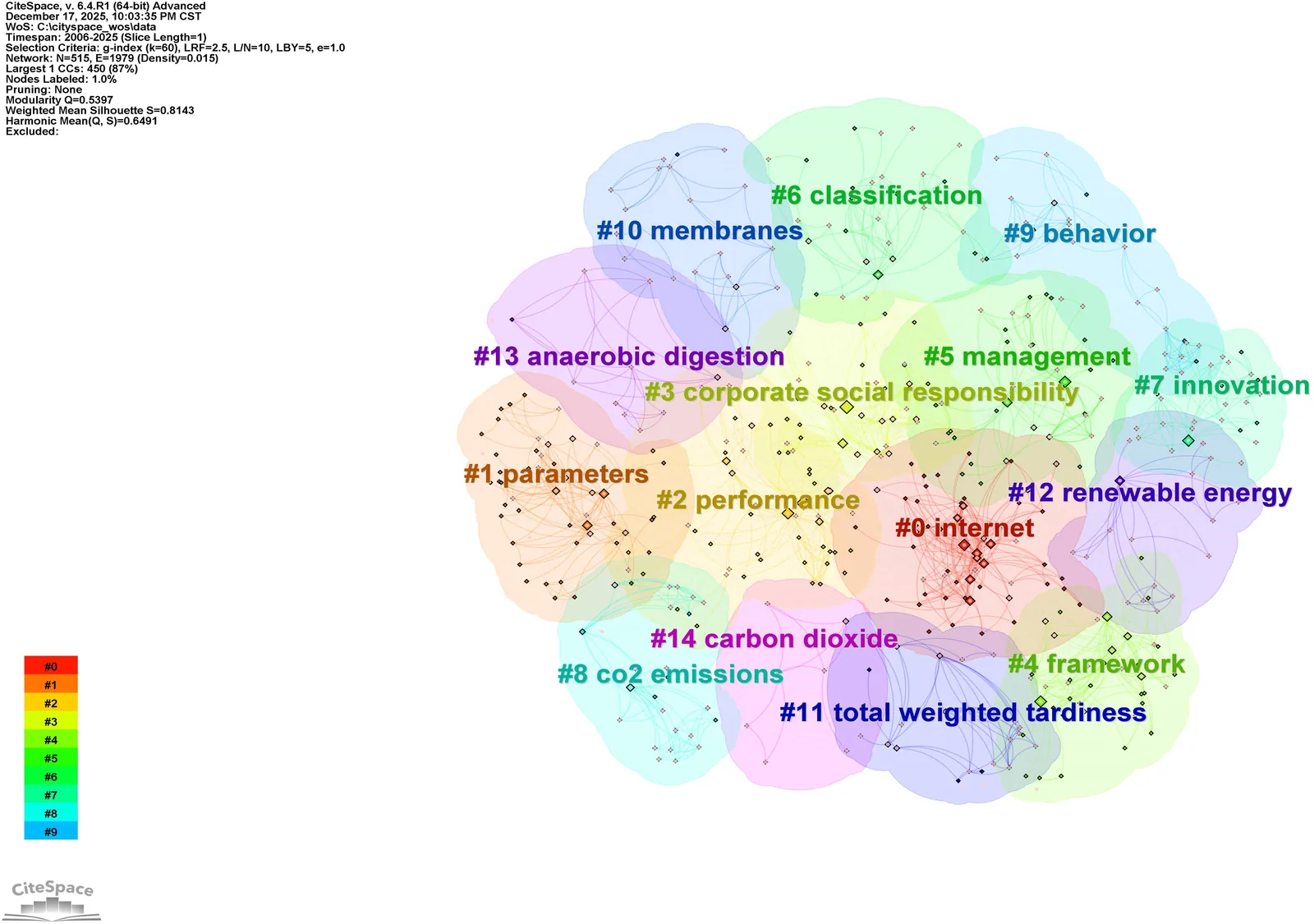
WOS English literature keyword clustering map.

The clustering results for the English-language literature (Fig 5) show a network modularity of Q = 0.5397 and a weighted average silhouette coefficient of S = 0.8143, indicating reliable clustering results. The analysis primarily formed thematic clusters such as “internet,” “performance,” “corporate social responsibility,” “management,” “innovation,” “CO_2_ emissions,” and “renewable energy,” covering areas including the application of digital technologies, corporate social responsibility, environmental performance, and energy transition. Although the research topics span multiple fields—including innovation, responsibility, and environmental governance—their common logic remains the view of digitalization as a tool for enhancing corporate capabilities and improving sustainability performance. The existing thematic framework has not yet fully revealed how digital resources achieve ESG value enhancement by transforming the corporate information environment and external governance relationships.

#### Literature review and the focus of this study.

The above analysis of the knowledge structure indicates that existing research has established a relatively clear framework oriented toward capability enhancement, namely, that digital resources primarily achieve corporate sustainability by promoting innovation capabilities, driving green innovation, and improving environmental performance. However, there remains room for expansion in two areas.

First, existing research primarily focuses on the technological transformation process through which digital transformation promotes green innovation, while paying insufficient attention to how digital resources create value in different industrial contexts. The production systems of energy-intensive enterprises are characterized by significant capital investment and path dependence; therefore, whether digital transformation can effectively improve ESG performance through traditional green innovation pathways requires further analysis in the context of specific industries.

Second, while existing research addresses topics such as information disclosure and corporate governance, it has not fully elucidated how digitalization influences ESG performance by improving the external information environment. Digital technologies not only transform internal production and innovation processes but also enhance information processing efficiency, improve information transmission mechanisms, and influence stakeholder perceptions; yet, this information governance mechanism remains under-researched.

In summary, the impact of digital transformation on ESG performance is not merely a single process of technological innovation, but rather the result of embedding digital resources into a firm's value creation system. This paper introduces the value-embedding model perspective, dividing the value realization process through which digitalization influences ESG performance into asset-embedding and information-embedding pathways. It then compares the mechanisms of these two pathways in the context of energy-intensive enterprises to reveal the intrinsic logic by which digital transformation drives improvements in corporate ESG performance.

## Theoretical framework and research hypotheses

### Theoretical foundation and analytical framework

The analysis in the preceding section indicates that the impact of digital transformation on corporate ESG performance is not a simple process of resource input leading to performance improvement; rather, its value realization may be jointly shaped by industry structural characteristics and resource conversion modes. On the one hand, asset rigidity in energy-intensive industries may alter the conditions under which digital resources are converted into ESG value; on the other hand, existing research still lacks a unified explanation of how digital resources unlock ESG value through different mechanisms. To this end, this paper adopts the Resource-Based View (RBV) as its overarching theoretical foundation, treating digital resources as strategic resources for enterprises to achieve sustainable development; further integrating Dynamic Capability Theory and Stakeholder Theory to explain, respectively, the two value-conversion mechanisms through which digital resources are transformed into green innovation and external information environments. Based on this, the paper constructs a theoretical analytical framework of “Resource-Based View—Value Conversion—ESG Performance,” providing a unified theoretical foundation for deriving research hypotheses in subsequent studies.

#### General theoretical foundation: The Resource-based view and the value of digital resources.

The resource-based view posits that a firm's sustainable competitive advantage stems from scarce, difficult-to-imitate, and irreplaceable strategic resources, while the realization of resource value depends on effective resource allocation and the transformation of capabilities [24]. With the deepening development of the digital economy, intangible assets such as data resources, digital technologies, and digital management capabilities have gradually become new strategic resources for enterprises [25–27], providing a new resource foundation for the green transition and sustainable development of energy-intensive industries.

Specifically in the context of energy-intensive industries, the value of digital resources does not stem from technological investment itself, but rather from their ability to provide differentiated pathways to overcome structural constraints in the industry's green transition: First, resources for precise energy consumption control rely on real-time data collection and intelligent analysis to improve energy efficiency and the controllability of production processes, thereby alleviating industry constraints such as high energy consumption intensity and high environmental governance costs [28]; Second, resources for collaborative resource allocation reduce coordination costs in the green transition of industrial chains by promoting information sharing and upstream-downstream optimization within supply chains, thereby alleviating the practical constraints faced by energy-intensive industries where emissions reductions rely on multi-stakeholder collaboration [29,30]; third, resources for information governance reduce stakeholders’ information acquisition costs by enhancing the standardization, visualization, and transmission efficiency of ESG information, thereby alleviating information asymmetry issues during environmental governance [31].

However, the resource-based view does not hold that strategic resources can automatically translate into competitive advantage; the realization of their value still depends on how they are integrated, allocated, and utilized. Furthermore, the resource orchestration perspective points out that the same resource base may lead to different outcomes in value creation depending on the mode of embedding and the organizational context. For digital resources, the mode of value embedding is determined by their target applications and the vehicles through which value is realized: digital resources aimed at optimizing production processes, technological upgrades, and enhancing industrial synergy require value transformation through adjustments to the enterprise's physical production system, forming an asset-embedded pathway; whereas digital resources aimed at enhancing information generation, transmission, and governance capabilities primarily achieve value creation by improving information relationships between the enterprise and external stakeholders, forming an information-embedded pathway.

Accordingly, there are at least two modes of value transformation for digital resources: one relies on resource integration and capability restructuring to foster green innovation and achieve technological empowerment; the other relies on the generation, transmission, and communication of information to improve the external information environment and achieve governance empowerment. Since these two pathways rely on adjustments to the physical production system and improvements in information governance capabilities, respectively—and thus have different foundations for value realization—the asset rigidity characteristic of energy-intensive industries may subject them to varying degrees of transformation constraints. This constitutes the core logical starting point for the comparison of the two pathways in this paper. The following sections will provide a detailed explanation of the intrinsic transformation mechanisms of these two pathways, drawing on dynamic capabilities theory and stakeholder theory, respectively.

#### Mechanisms of technology transfer: The theory of dynamic capabilities and pathways to green innovation.

The theory of dynamic capabilities posits that a firm's sustained competitive advantage stems not only from the possession of strategic resources but also depends on its ability to continuously integrate, reconfigure, and allocate resources to adapt to changes in the external environment [32,33]. This theory provides a core perspective for explaining the logic behind the transformation of digital resources into green innovation: the data resources, technological capabilities, and managerial capabilities generated by digital transformation do not automatically lead to green innovation; rather, they require a process of internal resource integration, capability restructuring, and innovative transformation within the enterprise to further yield substantive green innovation outcomes.

In the context of the green transition of energy-intensive enterprises, this dynamic capability is specifically manifested as “green dynamic capability”—that is, an enterprise's unique ability to integrate internal and external green resources, respond to environmental protection demands, and achieve green innovation [34]. Digital resources can enhance a company's environmental data processing capabilities, technology absorption capabilities, and R&D collaboration efficiency; reduce the costs of acquiring and applying green knowledge; promote the development of green products and the improvement of green processes; and ultimately systematically boost the company's green innovation output. Therefore, green innovation constitutes a key technological transformation pathway through which digital transformation influences ESG performance, and it is also the mechanism that has received the most attention in existing research.

However, it is important to note that dynamic capabilities theory primarily explains how firms develop and reconfigure green innovation capabilities; it does not guarantee that such capabilities will necessarily translate into improved ESG performance. For energy-intensive firms, green innovation ultimately requires value realization through tangible adjustments, including equipment upgrades, process optimization, and production system reforms, and therefore follows an asset-embedded value realization pathway. Given the structural characteristics of energy-intensive industries, such as capital intensity and long equipment replacement cycles, the transformation of green innovation outcomes into ESG value may involve substantial implementation and transformation costs, thereby limiting the effectiveness of this pathway under industry-specific constraints. Consequently, dynamic capabilities explain the underlying logic behind the formation of the technology-enabled pathway, while industry structural conditions define the boundaries of value realization through this pathway.

#### Governance-oriented transformation mechanism: Stakeholder theory and the external information environment pathway.

Stakeholder theory suggests that corporate sustainable development depends not only on economic performance but also on firms’ ability to respond to the diverse demands of stakeholders, including governments, investors, employees, consumers, and the broader public [35,36]. Enhancing organizational transparency and legitimacy through the continuous provision of accurate and comprehensive information constitutes an important foundation for firms to gain stakeholder trust and achieve long-term sustainable development.

For energy-intensive firms, the highly specialized, complex, and often opaque nature of environmental governance makes it difficult for external stakeholders to promptly and accurately assess firms’ actual environmental responsibility performance, thereby intensifying information asymmetry. Digital transformation improves the authenticity, completeness, and timeliness of environmental information through comprehensive data collection, real-time monitoring, and standardized information management. Meanwhile, digital platforms broaden communication channels between firms and stakeholders, reduce the costs associated with accessing ESG-related information, improve the external information environment.

Once the external information environment improves, its governance effects are primarily realized through two channels: On the one hand, a more transparent information environment enhances the efficiency of government regulation, capital market oversight, and public supervision, thereby strengthening corporate environmental responsibility and corporate governance practices and establishing a governance constraint mechanism; on the other hand, the improvement in the external information environment reduces the cost for stakeholders to identify companies’ actual responsibility-fulfillment behaviors, enabling ESG rating agencies, investors, and the general public to evaluate corporate sustainability performance more objectively, thereby reducing undervaluation caused by information asymmetry and forming an information identification mechanism [37]. Therefore, unlike green innovation pathways that rely on the restructuring of physical assets, the external information environment manifests as information-embedded value transformation, whose value realization depends primarily on improvements in information generation, transmission, and governance capabilities, rather than adjustments to the production system. Since this process involves minimal equipment upgrades and process modifications, the realization of its value is relatively less affected by the asset rigidity characteristic of energy-intensive industries.

### Research hypotheses

Based on the theoretical analysis above, this paper proposes the following research hypotheses, focusing on the overall effects of digital transformation and two value-conversion pathways: green innovation and the external information environment.

#### Digital transformation and corporate ESG performance.

The preceding analysis indicates that digital transformation may influence ESG performance by changing resource allocation and value realization. Therefore, this study examines the overall relationship between digital transformation and the ESG performance of energy-intensive firms.

Accordingly, the following research hypothesis is proposed:

H1: There is a positive relationship between digital transformation and the ESG performance of energy-intensive firms.

#### Green innovation as an asset-embedded path.

Digital transformation provides the foundational capabilities for corporate green innovation by enhancing the efficiency of resource integration, promoting the absorption of green knowledge, and strengthening R&D collaboration [38]. Green innovation has thus become a key technological pathway for digital resources to realize ESG value. However, green innovation is a typical asset-embedded value conversion pathway. Given the rigid constraints on assets in energy-intensive industries, converting digital resources into green innovation outcomes entails high transaction costs, and the ESG value of the technology-enabled pathway may be difficult to realize. Based on this, the following research hypotheses are proposed:

Based on this, the following research hypotheses are proposed:

H2: Green innovation is a key value-transformation pathway through which digital transformation influences the ESG performance of energy-intensive enterprises, but its effectiveness is constrained by the characteristics of assets in energy-intensive industries.

#### External information environment as an information-embedded path.

Unlike green innovation, which relies on adjustments to physical assets to achieve value transformation, the external information environment manifests as an information-embedded governance-driven empowerment pathway. As discussed in Section 3.1.3, digital transformation improves a company's external information environment and reduces stakeholders’ information identification costs, making the company's ESG performance easier for capital markets and other stakeholders to identify and evaluate. Since this pathway relies primarily on information-processing capabilities and governance coordination efficiency—rather than on adjustments to physical assets—its value realization is less constrained by the rigidity of assets in energy-intensive industries.

Based on this, the following research hypothesis is proposed:

H3: The external information environment mediates the relationship between digital transformation and the ESG performance of energy-intensive enterprises.

#### Differential effects of two value embedding paths.

Based on the theoretical analysis presented above, it can be observed that both pathways are important means by which digital resources realize ESG value, but the resource vehicles upon which the realization of value depends differ between the two. Accordingly, this paper proposes the analytical perspective of “Value Embedding Mode,” arguing that digital resources do not directly create ESG value on their own; rather, their role depends on the specific manner in which they are embedded into a company's value creation process. Specifically, green innovation falls under the “asset-embedded” pathway, in which digital resources are embedded within the production system to realize value; the realization of this value relies on the accumulation of physical capabilities such as green R&D, equipment upgrades, and production process improvements. The external information environment, on the other hand, falls under the “information-embedded” pathway, in which digital resources are embedded within the corporate governance process; the realization of its value primarily depends on improvements in information processing efficiency, information transparency, and governance coordination capabilities. Given the differences in the resource bases upon which these two types of value embedding rely, they may exhibit different levels of statistical stability in energy-intensive industries.

Based on this, the following research hypothesis is proposed:

H4: In the context of energy-intensive industries, the two transmission mechanisms through which digital transformation affects ESG performance exhibit different levels of value-conversion stability, with the information-embedded pathway being more stable than the asset-embedded pathway.

## Research design and empirical analysis

### Sample selection and data sources

The sample for this study focuses on A-share listed companies in energy-intensive industries, as defined by the National Development and Reform Commission's *Energy Efficiency Benchmark and Baseline Levels for Key Sectors in Energy-Intensive Industries*. The observation period was set from 2011 to 2024 to account for both the lag effects of digital transformation and the availability of continuous data. During the sample screening process, companies with financial anomalies classified as “ST” or “PT” were excluded in accordance with academic standards, and all continuous variables were trimmed at the 1st and 99th percentiles. This resulted in a total of 3,710 “firm-year” observations, of which 3,399 constituted the valid sample for the independent variable—the digital transformation index lagged by one period. All firm data were sourced from the CSMAR and CNRDS databases; missing data were supplemented and organized by reviewing the annual reports of listed companies.

### Variable definitions and measurement

1. Dependent Variable: Corporate ESG Performance
Drawing on Zhang Cheng's research [39], this study adopts the Huazheng ESG Rating as the metric for measuring corporate ESG performance. This rating system comprehensively evaluates companies across the three dimensions of environment, society, and governance. This study uses a composite ESG score ranging from 0 to 100, with higher scores indicating more effective fulfillment of corporate sustainability responsibilities. This serves as a precise measure of a company's ESG performance level.
2. Explanatory Variable: Level of Digital Transformation
This paper uses the “China Listed Companies Digital Transformation Index,” published by the School of Business at Shanghai University of Finance and Economics, to measure the level of digital transformation among enterprises. Based on textual data from listed companies’ annual reports, this index identifies content related to artificial intelligence, big data, cloud computing, blockchain, and the application of digital technologies by constructing a dictionary of digital keywords. It then quantifies the degree of digital transformation using text analysis and word frequency statistics. After standardization, a company-level digital transformation intensity indicator is derived; a higher index value indicates a higher level of digital transformation. Compared to text-based indicators developed independently by researchers, this index features a relatively mature construction framework and publicly available data sources, thereby enhancing the transparency of variable measurement and the reproducibility of research results.
3. Control Variables
Drawing on existing literature [40,41], this study further considers the following control variables: firm size (Size), debt-to-asset ratio (Lev), profitability (ROA), growth (Growth), and board independence (Board_Indep). Year and industry are treated as fixed-effects control variables. Specific variable definitions are provided in Table 1.
4. Mediating Variables
Green Innovation: As a core output resulting from enterprises’ integration of technological resources and response to environmental protection demands, green innovation is primarily manifested in creative practices such as R&D of environmental protection technologies, the iterative development of low-carbon products, and improvements to green processes. It serves as a key link connecting digital transformation with improvements in ESG performance. Drawing on the research of Zhang Hui [42] and others, this paper selects the annual number of green invention patent applications filed by firms in the CNRDS database as the core proxy indicator. The number of green innovation patent applications is increased by 1 and then log-transformed to approximate a normal distribution and improve the regression fit.
External Information Environment: Measured by the number of securities analyst teams that published research reports during the year; missing values are imputed as “0.” This indicator reflects the accessibility of a company's external information environment and the level of market attention it receives. It should be noted that analyst coverage is not information transparency itself, but rather an observable proxy for improvements in a company's external information environment. Digital transformation enhances the quality of information generation, improves the efficiency of information transmission, and reduces the cost of information acquisition for external parties. By increasing a firm's analyzability for information intermediaries, it may attract greater attention from analysts. Therefore, an increase in analyst coverage can serve as an important external indicator of improved corporate information transparency. Given that this indicator may still be influenced by factors such as firm size and market attention, this paper controls for relevant factors in the model and further discusses its limitations in Section 5.4.
5. Moderating Variables
Environmental Policy: A policy dummy variable was constructed using the announcement of the “Dual Carbon” goals as the cutoff point, with a value of 1 for 2021 and later, and 0 for earlier periods. This variable is used to capture the systemic impact of strengthened external regulatory requirements on firm behavior and serves as a moderating variable to test whether changes in the policy environment alter the strength of the association between digital transformation and ESG performance; growth potential (Tobin's Q) was included as a control variable in all regression analyses.
Ownership Structure: State-owned enterprises are assigned a value of 1, while private enterprises are assigned a value of 0 (Table 1).

**Table 1:** Variable definitions.

Variable Category	Variable Name	Symbol	Definition
**Dependent Variable**	Corporate ESG Performance	ESG	Composite ESG score ranging from 0 to 100
**Independent Variable**	Digital Transformation Level	DT_Index	China Listed Firms Digital Transformation Index published by the College of Business, Shanghai University of Finance and Economics
**Mediating Variable**	Green Innovation	Green_Innovation	Natural logarithm of (annual number of green invention patent applications + 1), denoted as ln(apply+1)
	External Information Environment	Transparency	Number of securities analyst teams releasing research reports in a fiscal year
**Control Variable**	Firm Size	Size	Natural logarithm of year-end total assets
	Leverage Ratio	Lev	Total liabilities / Total assets
	Profitability	ROA	Net profit / Total assets
	Growth Opportunity	Growth	Tobin's Q (obtained from the CSMAR database), calculated as market value / total assets
	Board Independence	Board_Indep	Number of independent directors divided by the total number of board directors
	Year	Year	Year fixed effect
	Industry	Industry	Industry fixed effect
**Moderating Variable**	Environmental Regulation	Policy	Dummy variable based on the “Dual Carbon” policy launched in 2021; equals 0 for years before 2021 and 1 for 2021 and subsequent years
	Ownership Nature	Ownership	Dummy variable; equals 1 for state-owned enterprises and 0 for private enterprises

### Model specification

To systematically test the research hypotheses, this study constructs the following empirical model framework.

#### Baseline regression model.

To test the positive relationship between digital transformation and the ESG performance of energy-intensive enterprises (H1), we employ mixed OLS regression, controlling for year and industry fixed effects, with standard errors clustered at the firm level. The full model includes all control variables; the baseline model (1) is specified as follows:


$$\begin{array}{c} Y_{it}=\beta_{\text{0}}+\beta_{\text{1}}X_{\_}lag_{it}+\beta_{\text{2}}K_{it}+\theta_{t}+\gamma_{j}+\varepsilon_{it} \end{array}$$ (1)

In the model, Yit denotes the dependent variable (ESG performance) for firm i in period t. The core independent variable $X\_lag_{it}$ represents the one-period lagged digital transformation index. $K_{it}$ includes a set of control variables: firm size (Size), leverage ratio (Lev), profitability (ROA), growth (Growth), and board independence (Board_Indep). $\theta_{\text{t}}$ and $\gamma_{\text{j}}$ indicate year fixed effects and industry fixed effects, respectively, and $\varepsilon_{\text{it}}$ is the random error term. If the estimated coefficient β_1_ is significantly positive, Hypothesis 1 is supported.

### Transmission effect models for the two mechanisms

1. Technology-Enabling Mechanism: Green Innovation Transmission Model (Tobit + OLS)
To test whether the technology-enabling mechanism corresponding to green innovation holds (H2), we use the coefficient product method (a × b) to test for indirect effects. Since the green innovation variable contains a large number of zero values (left-censored), we use a Tobit model to estimate path a to avoid OLS estimation bias; the remaining equations use OLS and control for year and industry fixed effects, with standard errors clustered at the firm level. The model is constructed as follows:
Path a (X_lag →Green_Innovation, left-censored at 0):

$$\begin{array}{c} Green\_Innovation_{it}^{*}=\alpha_{\text{1}}+\alpha_{\text{1}}X\_lag_{i,t-1}+Z_{it}\beta_{\text{1}}+Year_{t}+Indu_{j}+\varepsilon_{1it} \end{array}$$ (2)

$$\begin{array}{c} Green\_Innovation_{it}=max(Green\_Innovation_{it}^{*}) \end{array}$$

$Green\_Innovation_{it}^{*}$ denotes the latent variable, and $\varepsilon_{1it}\sim \;N\left(0,\sigma^{\text{2}}\right)$
Path b and Direct Effect (Impacts of X_lag and Green_Innovation on ESG):

$$\begin{array}{c} Y_{it}=\alpha_{\text{2}}+c_{\text{1}}^{\prime }X\_lag_{i,t-1}+b_{\text{1}}Green\_Innovation_{it}+Z_{\text{it}}\beta_{\text{2}}+Year_{t}+Indu_{j}+\epsilon_{2it} \end{array}$$ (3)

2. Governance Empowerment Mechanism: External Information Environment Transmission Model (Negative Binomial Regression + OLS)
To test whether the governance empowerment mechanism associated with the external information environment holds (H3)—given that the external information environment is a count variable with highly discrete characteristics—a negative binomial regression model is employed; ESG is treated as a continuous variable, and the path b and direct effects are estimated using ordinary least squares (OLS). All equations control for year fixed effects (Yeart), industry fixed effects (Induj), and a series of firm-level control variables ($Z_{it}$ including firm size (Size), debt-to-asset ratio (Lev), profitability (ROA), growth (Growth), independent directors (Board_Indep), environmental policy (Policy), and ownership structure (Ownership)), with standard errors clustered at the firm level. The model is constructed as follows:
Path a (X_lag →Transparency, count variable following negative binomial distribution):

$$\begin{array}{c} E(Transparency_{it}|\cdot )=𝐞𝐱𝐩(a_{3}+a_{2}X\_lag_{i,t-1}+Z_{it}\beta_{3}+Year_{t}+Indu_{j}) \end{array}$$ (4)

$$\begin{array}{c} Transparency_{it}\sim \;NegBin\left(\mu_{it},\theta \right) \end{array}$$

Due to the nonlinear nature of the model, we compute the average marginal effect (AME) of $X\_lag_{i,t-1}$ on $Transparency_{it}$, denoted as $a_{\text{2}}^{AME}$.
Path b and Direct Effect (Impacts of X_lag and Transparency on ESG):

$$\begin{array}{c} Y_{it}=\alpha_{\text{4}}+c_{\text{2}}^{\prime }X\_lag_{i,t-1}+b_{\text{2}}Transparency_{it}+Z_{\text{it}}\beta_{\text{4}}+Year_{t}+Indu_{j}+\epsilon_{3it} \end{array}$$ (5)

$\text{Indirect Effects}=a_{\text{2}}^{AME}\times b_{\text{2}}$, and standard errors are calculated via the Delta method.

#### Robustness test models.

To verify the reliability of the aforementioned regression results, this study designed the following robustness test models based on the empirical findings, including a model with individual fixed effects, nonlinearity tests, and endogeneity controls.

1. Individual Fixed-Effects Model

$$\begin{array}{c} Y_{it}=a_{i}+\beta_{1}X\_lag_{i,t-1}+\sum \gamma_{k}Control_{it}+\delta_{t}+\varepsilon_{it} \end{array}$$ (6)

$\alpha_{i}$ denotes the firm fixed effect, which captures time-invariant firm-level heterogeneity; $\delta_{t}$ represents the year fixed effect. $Control_{it}$ is consistent with the baseline model; standard errors are clustered at the firm level.
2. Nonlinearity Test (Quadratic Term Specification)
To test the potential nonlinear relationship, we add the quadratic term of X_lag to the baseline model:

$$\begin{array}{c} Y_{it}=\alpha +\beta_{1}X\_lag_{i,t-1}+\beta_{2}X\_lag_{i,t-1}^{2}+\sum \gamma_{k}Control_{it}+Year_{t}+Indu_{j}+\varepsilon_{it} \end{array}$$ (7)

$X\_lag_{i,t-1}^{\text{2}}$ denotes the quadratic term of the one-period lagged digital transformation index. $Control_{it}$ includes all control variables adopted in the baseline regression. We control for year fixed effects $Year_{t}$ and industry fixed effects $Indu_{j}$, with standard errors clustered at the firm level.
To mitigate multicollinearity, the quadratic term is constructed based on the centered X_lag (denoted as X_lag_c), and the estimation results remain equivalent.
3. Addressing Endogeneity
To alleviate potential endogeneity issues such as reverse causality and omitted variable bias between the one-period lagged digital transformation ($X\_lag_{it}$) and ESG performance ($Y_{it}$), we employ the instrumental variable approach, namely two-stage least squares (2SLS) estimation. Following existing studies (e.g., Fisman & Svensson [43]), we adopt the average value of X_lag for other firms within the same industry and year as the instrumental variable ($IV_{it}$).

The relevance of this instrument is based on the peer effect of digital transformation within the same industry, as firms’ digitalization processes may influence the digital transformation decisions of their industry peers. Considering that industry-level common factors may simultaneously affect firms’ ESG performance, this study further controls for industry and year fixed effects to mitigate the influence of industry-wide shocks. The instrumental variable results are therefore treated as supplementary evidence to address potential endogeneity concerns.

The model specifications are set out as follows:

First stage:


$$\begin{array}{c} X\_lag_{it}=\alpha_{0}+\alpha_{1}IV_{it}+\sum \gamma_{k}Control_{it}+\mu_{i}+\lambda_{t}+\varepsilon_{it} \end{array}$$ (9)

Second stage:


$$\begin{array}{c} Y_{it}=\beta_{0}+\beta_{1}\hat{X\_lag_{it}}+\sum \delta_{k}Control_{it}+\mu_{i}+\lambda_{t}+\nu_{it} \end{array}$$ (9)

where $IV_{it}$ denotes the instrumental variable (the average X_lag of other firms in the same industry); $\mu_{i}$ represents firm fixed effects; $\lambda_{t}$ denotes year fixed effects. Standard errors are clustered at the firm level.

### Analysis of empirical results

#### Descriptive statistics and correlation analysis.

The descriptive analysis (Table 2) systematically presents the basic distribution characteristics of the sample variables. The mean ESG score for the sample companies was 72.24, indicating that the overall ESG performance of energy-intensive enterprises has reached a certain level; however, the standard deviation of 5.972 suggests that significant differences in sustainable governance capabilities still exist among these companies. The mean Digital Transformation Index was 30.75, with a standard deviation of 6.194, reflecting significant variations in the degree of digital transformation among enterprises, as the accumulation of digital resources and the pace of transformation differ across companies. As two types of mechanism variables, green innovation had a mean of 0.418 and a standard deviation of 0.777, with a median of 0, indicating strong heterogeneity in the green innovation activities of energy-intensive enterprises; the external information environment had a mean of 5.362 and a standard deviation of 7.578, with the high degree of dispersion indicating significant differences in the extent of improvement in the information environment across enterprises.

**Table 2:** Descriptive analysis table.

Variable	N	Mean	SD	Min	p25	p50	p75	Max
**ESG**	3710	72.240	5.972	53.610	68.990	72.395	76.070	86.790
**DT_Index_lag1**	3399	30.750	6.194	23.226	26.310	28.526	34.655	49.382
**Size**	3710	23.021	1.485	19.653	21.984	22.920	24.004	26.710
**Lev**	3710	0.517	0.206	0.077	0.366	0.526	0.667	0.989
**ROA**	3710	0.027	0.060	−0.207	0.005	0.025	0.055	0.205
**Growth**	3710	1.504	0.906	0.774	1.030	1.216	1.591	6.473
**Board_Indep**	3710	0.374	0.052	0.333	0.333	0.333	0.429	0.556
**Green_Innovation**	3710	0.418	0.777	0.000	0.000	0.000	0.693	3.970
**Transparency**	3710	5.362	7.578	0.000	0.000	2.000	8.000	35.000
**Policy**	3710	0.329	0.470	0.000	0.000	0.000	1.000	1.000
**Ownership**	3710	0.550	0.498	0.000	0.000	1.000	1.000	1.000

Table 3 reports the correlation analysis among the main variables. The one-period lagged digital transformation variable (DT_Index_lag) is significantly and positively correlated with ESG performance (r = 0.144, p < 0.05), providing preliminary evidence for the subsequent regression analysis. Regarding the control variables, firm size, profitability, information transparency, and green innovation are positively associated with ESG performance, whereas firm growth and leverage exhibit negative correlations. Furthermore, both green innovation and information transparency are significantly correlated with digital transformation and ESG performance, providing preliminary support for the theoretical expectation that digital transformation influences ESG performance through technological embedding and governance embedding pathways. In addition, all variance inflation factor (VIF) values are below the critical threshold, indicating that there is no serious multicollinearity problem among the variables.

**Table 3:** Correlation analysis (n = 3,399).

	ESG	DT_Index_lag	Size	Lev	ROA	Growth	Board_Indep	Green_Innovation	Transparency	Policy	Ownership
**ESG**	1										
**DT_Index_lag**	0.144*	1									
**Size**	0.338*	0.288*	1								
**Lev**	−0.089*	−0.010	0.345*	1							
**ROA**	0.144*	0.027	0.030	−0.430*	1						
**Growth**	−0.217*	−0.163*	−0.539*	−0.168*	−0.022	1					

#### Benchmark regression results.

To test the relationship between digital transformation and the ESG performance of energy-intensive firms (H1), Table 4 presents the results of the baseline regression. After controlling for year fixed effects, industry fixed effects, and firm-level control variables, the coefficient for the digital transformation index (one period lagged) is 0.0467 (t = 1.50); the direction is positive but does not pass the conventional significance test; In a simplified model controlling only for year and industry fixed effects, the coefficient for digital transformation was 0.1349 and was significantly positive at the 1% level. These two sets of results indicate that digital transformation is generally positively associated with ESG performance, but this association weakens significantly when firm-level characteristics are included, suggesting that the ESG value generated by digital transformation may not be realized through a stable direct effect but rather relies on further transmission mechanisms.

**Table 4:** Regression results on the impact of digital transformation on the ESG performance of energy-intensive enterprises.

Variable	ESG	Green_Innovation	ESG	Transparency	ESG
	(1)	(2)	(3)	(4)	(5)
**DT_Index_lag1**	0.0467 (1.50)	0.0189 (1.94)*	0.0381 (1.25)	0.0407 (4.54)***	0.0952 (3.23)***
**Size**	1.6002 (9.30)**	0.8175 (11.66)**	1.4228 (7.41)**		
**Lev**	−6.5809 (−6.32)**	−0.6519 (−1.75)*	−6.0473 (−5.70)**		
**ROA**	2.6933 (1.06)	−1.9449 (−2.51)**	2.3861 (0.94)		
**Growth**	−0.1557 (−0.10)	0.0532 (0.84)	−0.1055 (−0.64)		
**Board_Indep**	5.1528 (1.55)	1.5737 (1.60)	4.7836 (1.44)		
**Policy**	−3.5304 (−5.82)**	–	–		
**Ownership**	1.0463 (2.27)*	–	–		
**Year/Industry**	Y	Y	Y		
**Green_Innovation**	–	–	0.7699(3.32)**		
**Transparency**	–	–	–		0.2051 (9.62)***
**Cons**	35.1805 (8.60)**	−20.2212 (−13.10)**	39.9439 (8.81)**	2.0871 (5.26)***	
**Rsquared**	0.2411	0.2094	0.2426		
**F**	13.80	13.28	14.30		

*Notes:* Standard errors are clustered by firm ID. Figures in parentheses are t-statistics. Cons denotes constant term. *p < 0.10, **p < 0.05, ***p < 0.01.

The results for the control variables were generally in line with expectations. Firm size significantly promotes ESG performance, indicating that larger firms possess a more robust resource base for implementing ESG practices; the debt-to-equity ratio was significantly negative, suggesting that higher financial constraints may limit a firm's capacity for sustainable investment; the effects of profitability and growth were not significant, indicating that short-term operating performance has limited explanatory power for ESG differences among energy-intensive firms.

#### Test results for the two transmission mechanisms.

Table 5 presents the results of the mediation analysis for the two types of transmission mechanisms. Based on the analytical framework proposed in Chapter 3, this study examines the mechanisms through which the technology pathway and the governance pathway influence the impact of digital transformation on ESG performance.

**Table 5:** Mediation effect estimation results.

Mediating Variable	Path & Effect Description	Effect Size	Standard Error	P-value	95% CI	Mediation Proportion (%)
**Green_Innovation**	Total effect of X_lag → Y (c)	0.1349	0.032	<0.001	[0.072, 0.198]	—
	Path (a): X_lag→Green_Innovation	0.0189	0.0097	P = 0.053	[-0.0002, 0.038]	—
	Path (b): Green_Innovation→Y	0.7599	0.2272	P = 0.001	[0.314, 1.226]	—
	Direct effect (c′)	0.0368	0.030	P = 0.209	[-0.022, 0.098]	—
	Indirect effect (a × b)	0.0143	0.0085	P = 0.094	[-0.0024, 0.0312]	10.6
**Transparency**	Total effect of X_lag → Y (c)	0.1349	0.032	<0.001	[0.072, 0.198]	—
	Path (a): X_lag→Transparency (AME)	0.2144	—	<0.001	—	—
	Path (b): Transparency→Y	0.205	0.021	<0.001	[0.163, 0.247]	—
	Direct effect (c′)	0.095	0.030	0.001	[0.036, 0.154]	—
	Indirect effect (a × b)	0.044	0.011	<0.001	[0.022, 0.066]	32.6

*Notes:* For Green_Innovation, Path (a) is estimated using a Tobit model (left-censored at 0), while Path (b) and the direct effect are estimated using OLS. For Transparency, Path (a) is estimated via negative binomial regression, and average marginal effects (AME) are calculated. Path (b) and the direct effect are estimated using OLS. The indirect effects are evaluated based on confidence intervals, and bootstrap resampling with 5,000 replications is conducted as a robustness check. The total effect is obtained from the baseline model excluding mediating variables (reg Y X_lag i.year i.Indu). Mediation proportion = (Indirect effect / Total effect) × 100%.

Regarding the technology-enabled mechanism, the estimated indirect effect of digital transformation through green innovation is positive but does not exhibit statistical robustness. Although Path a (digital transformation → green innovation) is marginally significant at the 10% level and Path b (green innovation → ESG performance) shows a significant positive effect, the confidence interval of the indirect effect includes zero. Therefore, based on the indirect-effect criterion, green innovation cannot yet be identified as a statistically robust transmission mechanism through which digital transformation affects ESG performance in the full sample. The bootstrap test further confirms this result: the confidence interval of the indirect effect still includes zero (Table 6), suggesting that the green innovation pathway does not constitute a statistically robust mediation channel in the full sample. These results suggest that digital transformation possesses the potential to promote green innovation, but the further translation of this potential into ESG value remains subject to industry-specific conditions. Specifically, the outcomes of green innovation must undergo adjustments to production systems and the integration of organizational resources before they can ultimately manifest as improvements in ESG performance; factors such as capital-intensive characteristics and technology upgrade cycles may increase the difficulty of achieving this transformation.

**Table 6:** Bootstrap test of indirect effects for the two transmission mechanisms.

Mediating Variable	Indirect Effect (a × b)	Bootstrap SE	z-value	p-value	95% Bootstrap CI
**Green_Innovation**	0.01334	0.00913	1.46	0.144	[-0.00456, 0.03123]
**ln(Transparency)**	0.00173	0.00046	3.77	0.000	[0.00083, 0.00262]

*Notes:* This table reports the bootstrap robustness test of the indirect effects. The bootstrap procedure resamples firms with replacement and re-estimates the mediation models. The procedure is based on 5,000 replications. For the Transparency mechanism, ln(Transparency) is used as the mediator in the bootstrap estimation to improve distributional properties and estimation stability during resampling. Confidence intervals are based on bootstrap standard errors.

In contrast, the governance-empowerment pathway exhibits more stable statistical characteristics. Digital transformation significantly improves a firm's external information environment and further promotes enhanced ESG performance, with the mediating effect accounting for 32.6% of the total effect. Bootstrap resampling results further show that the Bootstrap confidence interval for the indirect effect of this pathway does not cross zero, indicating that improvements in the external information environment constitute a key transmission channel through which digital transformation influences ESG performance. These results indicate that digital transformation not only enhances a firm's technological capabilities but also facilitates the realization of governance value by improving the processes of information generation, transmission, and external communication. Since this pathway primarily relies on improvements in information processing capabilities and governance coordination efficiency—and involves relatively few adjustments to physical assets—it demonstrates strong adaptability in the context of energy-intensive industries.

Overall, the two transmission mechanisms exhibit distinct statistical stability characteristics: the green innovation pathway is subject to stronger constraints associated with physical production-system adjustments and does not exhibit a statistically robust transmission effect; in contrast, the external information environment pathway generates a significant mediating effect and demonstrates greater stability. The above conclusions remain valid under bootstrap resampling tests, further enhancing the reliability of the mechanism identification results. This finding supports the analytical framework of “value embedding modes” proposed in this paper, namely, that the ESG value of digital resources depends on the specific manner in which they are embedded in the firm's value creation process.

#### Robustness tests.

1. Firm-Specific Fixed Effects Model

As shown in column (6) of Table 7, after controlling for firm-specific fixed effects, the coefficient for digital transformation is 0.0333. Although it remains positive, it does not pass the significance test. This result indicates that, after further controlling for firm-specific characteristics that do not change over time, the direct impact of digital transformation on ESG performance still does not exhibit a consistent statistical effect, consistent with the conclusion from the baseline regression that “digital transformation unleashes ESG value through internal transmission mechanisms.”

**Table 7:** Summary of Robustness Test Results.

Variable	ESG	ESG
	(6)	(7)
**X_lag_c**	0.0333 (1.00)	0.0039 (0.10)
**X_lag_c^2^**	–	0.0065 (1.87)*
**Size**	1.4286 (5.11)***	1.6095 (9.34)***
**Lev**	−4.6942 (−4.19)***	−6.5452 (−6.28)***
**ROA**	−1.0398 (−0.48)	2.9275 (1.17)
**Growth**	0.1695 (0.97)	−0.311 (−0.20)
**Board_Indep**	7.9118 (2.44)**	4.9438 (1.47)
**Policy**	0 (omitted)	0 (omitted)
**Ownership**	0.9689 (1.24)	1.0654 (2.33)**
**Year/Industry**	Y/-	Y
**Con**	36.9133***	35.7276***
**R^2^**	0.4979	0.2431
**F**	6.01	25.29

*Notes:* Clustered SEs by firm ID. Values in parentheses are t-values; “Cons” denotes the constant term. * p < 0.10, ** p < 0.05, *** p < 0.01

1. Nonlinear (Squared Term) Test

To further examine whether a nonlinear relationship exists between digital transformation and ESG performance, this study incorporates a squared term for digital transformation (constructed based on the centered variable) into the baseline model. As shown in column (7) of Table 6, the coefficient of the squared term for digital transformation is 0.0065 (t = 1.87), which is marginally significant at the 10% level, indicating that the impact of digital transformation on ESG performance is not entirely linear.

**Fig 6:**
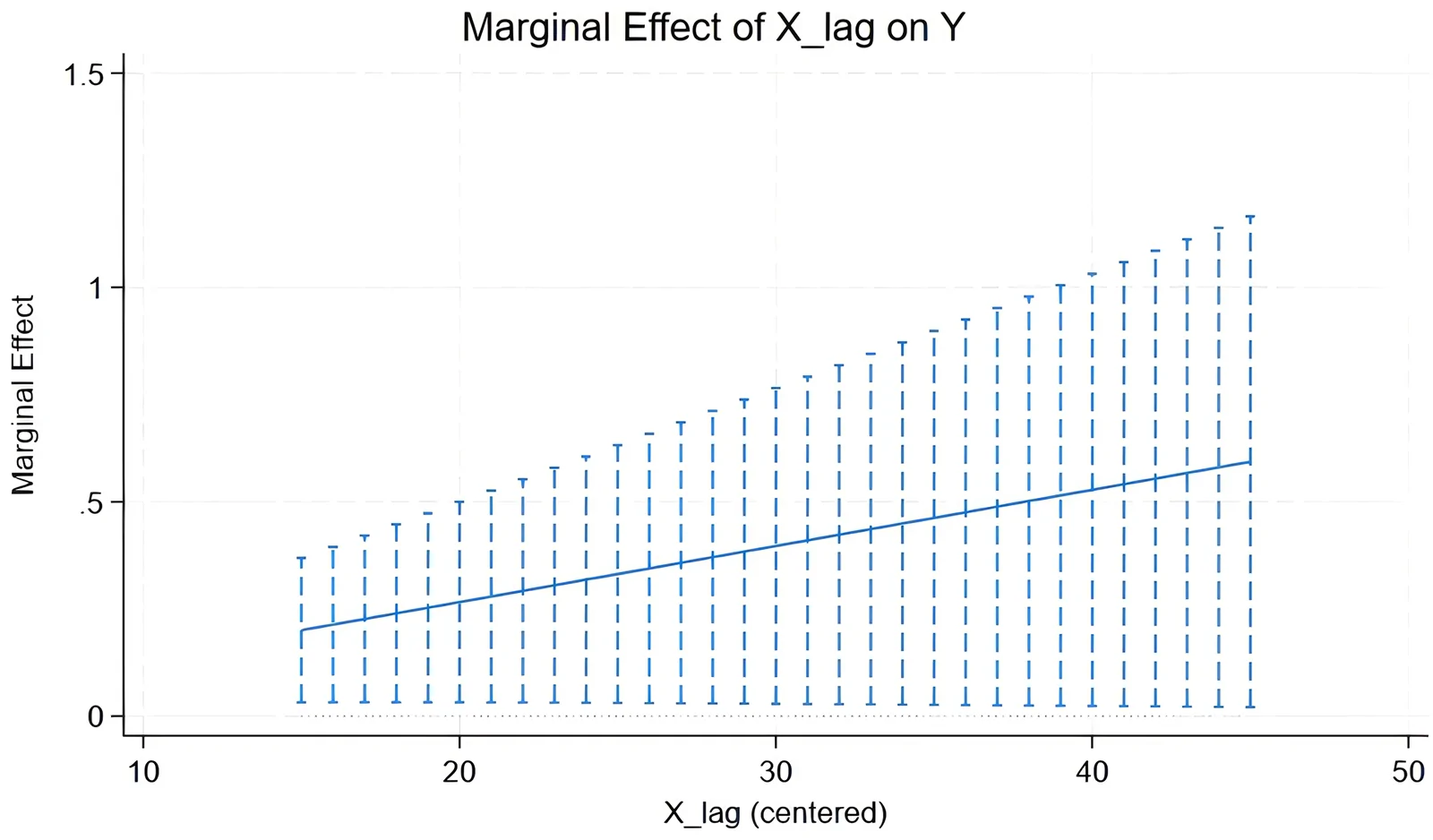
Marginal effects of digital transformation on ESG performance.

Further analysis of the marginal effect results in Fig 6 reveals that the marginal effect of digital transformation on ESG performance remains consistently positive across the sample range and exhibits an increasing trend as the level of digitalization rises. This implies that there is no distinct inflection point where digital transformation shifts from a negative constraint to a positive driver; rather, the release of its ESG value gradually intensifies as digital resources accumulate and organizational capabilities deepen. In other words, the impact of digital transformation does not depend on a single technological investment, but rather on whether digital resources can be continuously embedded into corporate governance and operational processes to generate cumulative value creation.

This finding further complements the results of the benchmark regression and mechanism tests: although digital transformation does not exhibit a stable direct effect on ESG, its value is not nonexistent; rather, it needs to be gradually unleashed through the restructuring of internal capabilities and the refinement of governance mechanisms. It should be noted that, since the squared term reached only marginal significance and the marginal effects plot reflects trends within the sample range, this study does not interpret these findings as a strict U-shaped relationship or a clear threshold effect, but rather regards them as supporting evidence for the gradual release of digital value.

1. Treatment of Endogeneity

Given the potential for a reverse causal relationship between digital transformation and corporate ESG performance, this paper employs the instrumental variables method to test for endogeneity (Table 8). Specifically, we select the mean digital transformation level of other firms in the same industry as the instrumental variable (IV_IndustryMean) to reflect the influence of the digital diffusion environment within the firm's industry on its own digital transformation decisions, while controlling for industry and year fixed effects.

**Table 8:** Impact of being one phase behind in digital transformation on ESG (N = 3,390).

Variable	Phase 1	Phase 2
**IV_IndustryMean**	0.377 (0.086)***	
**Digital Transformation Lag (Phase 1) (Fitted Values)**		0.263 (0.263)
**Size**	1.868 (0.292)***	0.885 (0.562)
**Lev**	−3.700 (0.975)***	−3.785 (1.325)***
**ROA**	−2.099 (1.746)	−0.810 (2.206)
**Growth**	0.236 (0.127)*	0.081 (0.195)
**Board_Indep**	6.430 (2.304)***	6.255 (3.707)*
**Green_Innovation**	0.558 (0.178)***	0.152 (0.297)
**Transparency**	−0.047 (0.025)*	0.038 (0.026)
**Policy**	omitted	omitted
**Ownership**	0.221 (0.937)	0.890 (0.848)
**Year/Industry**	Y	Y
**R^2^**	–	0.004
**Phase 1 F-Statistic**	18.80***	
**Kleibergen-Paap rk LM**	17.98***	
**Cragg-Donald Wald F**	91.34	
**Stock-Yogo 10% Threshold**	16.38	

*Notes:* Figures in parentheses represent cluster-robust standard errors (clustered at the firm level). ** p < 0.01, ** p < 0.05, * p < 0.1. *

The results of the first stage show that the instrument coefficient is 0.377, which is significantly positive at the 1% level, indicating that the digitalization level of other firms in the same industry effectively explains the firm's own degree of digital transformation. The Kleibergen-Paap rk LM statistic is 17.98 and is significant at the 1% level; the F-statistic for Stage 1 is 18.80, which is higher than the Stock-Yogo 10% critical value of 16.38, indicating that there is no significant weak instrument problem.

The results of the second stage show that the coefficient for digital transformation remains positive but does not reach statistical significance. This indicates that, after correcting for potential endogeneity biases using the instrumental variables method, the direct impact of digital transformation on ESG performance still does not exhibit stable and significant characteristics, consistent with the findings from the baseline regression that the direct effect of digital transformation is weak. Further analysis of the mediation effect test results suggests that the impact of digital transformation on ESG performance may rely more on transmission mechanisms such as green innovation and the external information environment, rather than manifesting as a stable direct effect.

### Research conclusions and outlook

#### Key research conclusions.

The core conclusion of this paper can be summarized as follows: The ESG value of digital resources is not directly determined by the scale of investment, but rather depends on the specific manner in which value is embedded; the asset rigidity in energy-intensive industries imposes asymmetric constraints on the asset-embedded (green innovation) and information-embedded (external information environment) pathways, ultimately resulting in a divergent pattern characterized by “technological pathways constrained by asset rigidity, while governance pathways exhibit greater stability.”

Specifically, there is a positive association between digital transformation and the ESG performance of energy-intensive enterprises, but no stable direct effect. Digital resources cannot automatically translate into ESG performance; the realization of their value must rely on specific transmission mechanisms. Further analysis reveals that the asset-embedded pathway corresponding to green innovation has not established a stable transmission mechanism. Constrained by structural characteristics such as capital intensity and high asset specificity, the efficiency of converting digital resources into green innovation is weakened by asset rigidity, making it difficult to effectively unlock the value of technological empowerment.

In contrast, the information-embedded pathway—corresponding to the external information environment—serves as a robust transmission channel. Digital technology reduces information asymmetry by optimizing mechanisms for information generation, transmission, and oversight, thereby driving ESG improvements through governance-enabled approaches; compared to the green innovation pathway constrained by asset rigidity, the realization of its value does not depend on adjustments to physical assets, resulting in greater transmission stability. Thus, the core difference between the two pathways lies not in the magnitude of their effects, but in their statistical stability under highly constrained conditions. The information-embedded pathway exhibits greater resistance to constraints and represents a more reliable means of realizing digital ESG value for energy-intensive enterprises.

#### Theoretical contributions.

First, this paper proposes an analytical framework of “value embedding modes,” thereby expanding the explanation of the mechanisms through which digital transformation influences ESG performance. Existing research largely remains at the level of assessing effects, failing to open the “black box” of value transformation from digital resources; moreover, mechanism studies generally focus on the magnitude of effects while neglecting contextual stability. This paper distinguishes between two value-conversion models—asset embedding and information embedding—and demonstrates that they exhibit divergent performance under asset rigidity constraints. This not only provides a new explanatory tool for the heterogeneity of digital sustainable value but also broadens the perspective of mechanism research from “effect strength” to “path stability.”

Second, this paper reexamines the applicability of green innovation as the core mechanism through which digitalization empowers ESG. While existing literature, largely based on cross-industry samples, assumes green innovation to be the core transmission mechanism, this study—using a sample from energy-intensive industries—demonstrates that asset rigidity significantly weakens the conversion efficiency of technological pathways, indicating that technology-enabled pathways are not consistently stable or effective across all industrial contexts.

#### Policy implications.

At the corporate level, energy-intensive enterprises should select a digital ESG transformation path suited to the inflexible nature of their assets. For traditional manufacturing enterprises with highly specialized assets and long retrofitting cycles for existing equipment, the short-term priority should be to leverage digital technologies to enhance energy consumption monitoring, environmental data collection, and ESG disclosure capabilities. By empowering governance, they can unlock the value of digitalization and avoid blindly pursuing high-cost technological upgrades when conditions are not yet ripe. For enterprises with significant room for asset renewal and the conditions for production capacity upgrades, digital technologies can be further integrated into green R&D and production processes in conjunction with equipment upgrades and process optimization, thereby achieving synergistic progress in both technological empowerment and governance enhancement.

At the industrial policy level, support mechanisms for green innovation should be refined to address the transformation constraints faced by industries with high asset rigidity. For industries such as steel, cement, and coking—where equipment retrofitting costs are high—policy support should not adopt a one-size-fits-all digital assessment model. Instead, it should reduce the costs of adjusting physical assets through targeted support for digital green technological upgrades, financing facilitation, and tax incentives, thereby increasing the feasibility of converting digital resources into green innovation. At the same time, avoid imposing technological transformation requirements on existing enterprises that exceed their transformation capabilities to prevent a mismatch between policy objectives and the actual capacity of enterprises.

At the ESG regulatory level, a tiered disclosure mechanism aligned with enterprises’ information governance foundations should be further refined. For enterprises with strong digital foundations and favorable external information environments, verifiable digital ESG disclosure requirements should be implemented first, mandating the integration of operational condition monitoring and emissions network data to amplify the effect of governance constraints. For small and medium-sized enterprises (SMEs) with weak information foundations, support should be provided through industry-standardized disclosure templates and lightweight energy-and-carbon management SaaS tools to gradually improve information governance levels and unlock the inclusive value of digital governance.

#### Research limitations and prospects.

First, in terms of variable measurement, this paper still has certain limitations. Constrained by data availability, this paper uses analyst coverage and patent counts to measure the external information environment and green innovation level, respectively. Although these proxies can, to some extent, characterize the relevant mechanisms, they still cannot fully capture the dynamic changes in firms’ information governance capabilities and innovation quality. Future research could combine ESG disclosure text analysis, patent quality evaluation, and data on firms’ digital application scenarios to further improve the precision of variable measurement.

Second, in terms of causal identification, there remains room for further deepening. Although this paper enhances the reliability of its conclusions through multiple empirical methods, endogeneity issues may still exist between digital transformation and firms’ ESG performance. Future research could leverage quasi-natural experimental designs such as digital economy pilot programs and mandatory ESG disclosure policies to obtain stronger causal identification evidence.

Finally, in terms of expanding the theoretical framework, the analysis of this paper still has certain boundaries. This paper primarily focuses on two typical value realization pathways—asset embeddedness and information embeddedness—and has not yet covered other possible ways through which digital resources affect ESG performance. Subsequent research could explore more models such as supply chain embeddedness and organizational embeddedness, and conduct cross-industry comparisons based on the asset rigidity characteristics of different industries, in order to refine the theoretical explanatory framework for how digital transformation promotes sustainable development.
