# Supplementary material for: Beyond digital investment: How value embedding modes shape ESG performance in energy-intensive firms
Source: PLoS One. 2026 Sep 21;21(9):e0343345. doi: 10.1371/journal.pone.0343345 (PMC13592625; doi:10.1371/journal.pone.0343345)
Supplement: Supplementary file 1 [file pone.0343345.s001.docx]

This paper examines how energy-intensive enterprises can enhance their ESG performance through digital transformation in the context of China's "dual carbon" goals. It constructs a theoretical model that incorporates green innovation as a mediating variable and corporate growth potential as a moderating variable, using a mixed-method approach that combines bibliometric analysis with empirical testing.

The following suggestions are offered for your reference:

**The Literature Review Section is Somewhat Repetitive and Could Be Condensed**
The descriptions in Section 2 regarding "Knowledge Structure" and "Frontier Evolution" contain some redundancy. It is recommended to merge or streamline these parts, highlighting core findings rather than interpreting each figure sequentially.

**The Measurement of Key Variables Needs Further Clarification**
The "Digital Transformation Index" is sourced from Shanghai University of Finance and Economics. It would be helpful to briefly explain its construction method (e.g., keyword extraction logic, weighting scheme) to enhance transparency and replicability.

**The Measurement of Green Innovation Suffers from a "Zero Inflation" Problem**
With over 50% of firms having zero green innovation output, this could affect the robustness of the regression results. The authors should consider addressing this, for instance by employing a Zero-Inflated model or a Tobit model as a robustness check, or at least discussing this limitation.

**The Explanation for the Negative Mediating Effect in the High-Growth Group is Insufficient**
The negative mediating effect in the high-growth group is attributed to "resource crowding out." However, theoretically, it could also be due to a "lag effect" where digital investments have not yet yielded green outcomes. A more in-depth discussion or analysis using lag structures might be beneficial.

**The Policy Implications are Somewhat Generic and Could be More Targeted**
The policy recommendations are broad. It is suggested to make them more specific based on the empirical results. For example, propose green innovation subsidies for low-growth firms and stricter ESG disclosure requirements for high-growth firms.

**Figure and Table Numbering is Inconsistent, with Citation Errors**
The manuscript contains multiple instances of "Error! Reference source not found." throughout the text. A thorough proofread to correct all figure/table cross-references is necessary.

**The Language is Occasionally Redundant and Could Benefit from Polishing**
Some sections, particularly in the theory-building part, contain repetitive phrasing. Streamlining the language would improve readability and conciseness.
