# Supplementary material for: Beyond digital investment: How value embedding modes shape ESG performance in energy-intensive firms
Source: PLoS One. 2026 Sep 21;21(9):e0343345. doi: 10.1371/journal.pone.0343345 (PMC13592625; doi:10.1371/journal.pone.0343345)
Supplement: Supplementary file 2 [file pone.0343345.s002.docx]

**Reviewer Recommendation: Major Revision**

The manuscript investigates the relationship between digital transformation and ESG performance in Chinese energy-intensive enterprises, exploring the mediating role of green innovation and the moderating effect of growth potential. While the research context (China's "dual carbon" goals) is highly relevant and the inclusion of growth potential as a negative moderator provides an interesting theoretical angle, the manuscript currently suffers from severe methodological flaws and a significant structural imbalance. To meet PLOS ONE’s rigorous criteria for statistical robustness and methodological validity, substantial revisions are required.

Below are the major and minor concerns that need to be addressed:

**Majors**

**-** The authors measure the mediating variable, "Green Innovation," as the natural logarithm of the number of green invention patent applications plus one. The descriptive statistics reveal that the median is 0, indicating that a significant portion of the sample has zero patent applications. Applying standard Ordinary Least Squares (OLS) regressions to count data with a heavy right skew and zero-inflation violates fundamental statistical assumptions (normality and homoscedasticity of residuals), leading to biased and inconsistent estimates.

- Need to address 🡪 The authors must re-estimate the models predicting green innovation using models appropriate for count or censored data, such as a Poisson regression, Negative Binomial regression, or a Tobit model.

The study uses panel data and claims a "direct impact" of digital transformation on ESG performance. To mitigate reverse causality, the independent variable is lagged by one period. In corporate finance and management research, a simple one-year lag is insufficient to resolve endogeneity concerns. Firms with stronger ESG performance often have better access to financing, allowing them to invest more heavily in digital transformation (simultaneity/reverse causality). Furthermore, the risk of omitted variable bias remains high.

- Need to address 🡪 The authors must implement a more robust identification strategy to address endogeneity. Acceptable approaches include Instrumental Variables (IV/2SLS), Generalized Method of Moments (GMM), or Propensity Score Matching with Difference-in-Differences (PSM-DID). Without these robustness checks, the paper can only claim correlations, not causal impacts.

The manuscript relies on the Baron and Kenny (1986) causal steps approach and the Sobel test to establish mediation. The Baron and Kenny method is widely considered outdated in modern econometrics and psychology, as it suffers from low statistical power. Similarly, the Sobel test assumes a normal distribution for the indirect effect, which is rarely the case.

**-** Need to address 🡪 The authors should use a Bootstrapping approach (e.g., using the PROCESS macro or Structural Equation Modeling) to test the significance of the indirect effect for the entire mediation model, replacing the Baron and Kenny framework entirely.

A disproportionate amount of the manuscript is dedicated to a CiteSpace bibliometric analysis simply to justify the choice of variables and identify the research gap. This creates a structural imbalance, making the manuscript read like two separate, disjointed papers. In an empirical financial/management paper, the research gap should be established through a concise, well-written literature review, not extensive keyword network mappings.

**-** Need to address 🡪 The bibliometric section should be drastically condensed (to 2-3 pages maximum) or moved entirely to the Supplementary Materials/Appendix. The core focus of the manuscript must be the econometric model, its robustness, and the economic interpretation of the findings.

**Minors**

- The authors state that the raw data were obtained from third-party databases (CSMAR and CNRDS). While providing links to these databases is helpful, PLOS ONE has a strict open-data policy.

- Need to address 🡪 To ensure perfect replicability without violating third-party copyright of the raw databases, the authors should upload their final, compiled, and cleaned dataset (e.g., in .csv or .dta format) along with the replication code (Stata do-files or R scripts) as Supporting Information.

**-** Several figures generated via CiteSpace suffer from poor resolution, overlapping text, and a confusing mix of English and Chinese characters.

- Need to address 🡪 All figures must be redesigned or exported at a higher resolution (minimum 300 dpi) to ensure all node labels are legible and clearly translated into English, in accordance with the journal's formatting guidelines.

**-** The manuscript occasionally uses hyperbolic language (e.g., "unprecedented," "exponential growth," "profoundly reflects").

- Need to address 🡪 Please review the manuscript to ensure a more objective, neutral, and scientifically rigorous tone throughout the text. Let the data and the statistical significance speak for the magnitude of the findings.
