# Supplementary material for: Beyond digital investment: How value embedding modes shape ESG performance in energy-intensive firms
Source: PLoS One. 2026 Sep 21;21(9):e0343345. doi: 10.1371/journal.pone.0343345 (PMC13592625; doi:10.1371/journal.pone.0343345)
Supplement: Supplementary file 3 [file pone.0343345.s004.docx]

**Response to Reviewers**

**Manuscript ID:** PONE-D-26-05685

**Original title:** Digital Transformation and ESG Performance in Energy-Intensive Enterprises: The Mediating Role of Green Innovation and the Moderating Effect of Growth Potential

**Revised title:** Beyond Digital Investment: How Value Embedding Modes Shape ESG Performance in Energy-Intensive Firms

**Author:** Weiran Sun (School of Management, Nanjing University of Posts and Telecommunications)

**Journal:** PLOS ONE

## **1. Note on the scope of this revision**

Given the extent of the revisions, I first summarize the main changes that guided the restructuring of the manuscript.

Reviewer 2’s Major Comments 1 and 3 prompted two important methodological revisions: first, reconsidering the estimation approach for the green innovation mechanism given the large proportion of zero observations; and second, replacing the original mediation testing procedure with a more appropriate indirect-effect assessment framework.

After re-estimating the green innovation pathway using a Tobit model and evaluating the indirect effect based on confidence intervals, the evidence supporting green innovation as a stable transmission mechanism became weaker than under the original specification. Specifically, although digital transformation remains positively associated with green innovation (Path a: coefficient = 0.0189, p = 0.053), and green innovation is significantly associated with ESG performance (Path b: coefficient = 0.7599, p = 0.001), the indirect effect does not achieve statistical robustness because its 95% confidence interval includes zero (indirect effect = 0.0143, p = 0.094). Bootstrap estimation further confirms this result. Therefore, the revised manuscript no longer claims that green innovation constitutes a statistically robust mediation pathway through which digital transformation improves ESG performance.

The original moderated mediation analysis based on the green innovation pathway was also removed because its theoretical interpretation depended on a transmission mechanism that was not sufficiently supported after the revised estimation.

Rather than retaining conclusions that were not supported by the updated empirical evidence, I further examined why the technology-oriented pathway may face limitations in energy-intensive industries and reconsidered the broader mechanisms through which digital resources generate ESG value. Specifically, I conducted additional literature analysis and revisited the bibliometric evidence, including keyword co-occurrence and clustering patterns, together with an expanded review of both international and Chinese studies. This process revealed that existing research has been predominantly concentrated on the technology-oriented pathway, particularly the role of digital transformation in promoting green innovation, while the governance-oriented pathway through which digital resources improve information-related capabilities has received comparatively less attention.

Based on this theoretical reconsideration, the revised manuscript incorporates the external information environment as an additional information-embedded pathway through which digital resources may generate ESG value. This revision extends the analytical framework to capture alternative modes through which digital resources can be embedded into corporate value creation processes. The original green innovation pathway is retained in the empirical analysis (with improved estimation), while the information-environment pathway is added as a complementary mechanism. The revised framework therefore does not discard the original mechanism, but situates it within a broader theoretical structure that distinguishes between asset-embedded and information-embedded value conversion.

Because the external information environment is measured as a count variable with over-dispersion, the revised analysis estimates this transmission pathway using negative binomial regression with average marginal effects. The results provide evidence for an information-embedded mechanism: digital transformation significantly improves the external information environment (Path a: AME = 0.2144, p < 0.001), which subsequently enhances ESG performance (Path b: coefficient = 0.205, p < 0.001). The indirect effect is statistically significant (effect = 0.044, p < 0.001, 95% CI [0.022, 0.066]), and the result remains supported by bootstrap confidence intervals.

Based on these revisions, the manuscript has been reframed from a single-mechanism model with growth moderation into a “value embedding mode” framework. Specifically, the revised study examines two pathways through which digital resources may be converted into ESG value: an asset-embedded pathway represented by green innovation and an information-embedded pathway represented by the external information environment.

The revised theoretical framework further explains why these two pathways may exhibit different levels of effectiveness in energy-intensive industries. Due to characteristics such as capital intensity, asset specificity, and long equipment replacement cycles, asset rigidity may increase the difficulty of embedding digital resources into physical production systems and realizing green innovation outcomes. In contrast, information-based value creation relies more on improvements in information processing, communication, and governance coordination capabilities, and is therefore relatively less dependent on physical asset adjustment.

Accordingly, the title, abstract, introduction, theoretical framework, hypotheses, empirical models, results, and conclusions have all been revised. Firm growth potential (Tobin’s Q) is retained as a control variable rather than a moderating variable, and the previous resource-crowding-out explanation has been removed.

# **2. Response to Reviewer 1**

### **Comment 1.1 — The literature review is somewhat repetitive and could be condensed**

**Reviewer comment:** "The Literature Review Section is Somewhat Repetitive and Could Be Condensed — The descriptions in Section 2 regarding 'Knowledge Structure' and 'Frontier Evolution' contain some redundancy. It is recommended to merge or streamline these parts, highlighting core findings rather than interpreting each figure sequentially."

**Text in the original submission:** "The keyword co-occurrence analysis shows (Figures 2 and 3) that both Chinese and English literature have formed a strongly consensus-based pathway of 'digital transformation -- green innovation -- ESG', which constitutes the core association hub and high-density association chains. This highly convergent knowledge network, rather than being a summary of underlying laws, more closely resembles an academic 'path lock-in'—where the research perspective is anchored onto this main track, invisibly delineating the boundaries of discourse. ... This absence is not a coincidental omission but profoundly reflects the 'systemic neglect' of the field, when constructing a universal explanatory framework, towards the industry subjects with the most acute transformation challenges and their internal dynamic developmental factors."

(Section "Knowledge Structure: How Core Consensus Obscures the Specificity of Energy-Intensive Enterprises", p. 13)

"In summary, these three discontinuities collectively reveal a fundamental shortcoming: the field has yet to provide an integrated analytical framework that focuses on the specific context of energy-intensive enterprises, bridges the logic of 'external pressures and internal capabilities', and uncovers micro-regulatory mechanisms for growth. Consequently, this study will directly address this deficiency in Chapter Three..."

(Section "Bibliometric Summary: Three Research Gaps Pointing to energy-intensive enterprises", p. 22）

**My response and how the manuscript was revised:**

I appreciate this valuable suggestion. And I acknowledge that the previous version provided relatively detailed interpretations of each bibliometric analysis, which resulted in some overlap among the discussions of knowledge structure, keyword clustering, and research gaps. I made the following revisions:

1.The three interpretive subsections of the original Section 2 (2.4 Knowledge Structure; 2.5 Keyword Cluster Analysis; 2.6 Bibliometric Summary) Ire merged into a single section, “Literature Review and Identification of Research Gaps”, containing two short subsections plus one closing synthesis. At the same time, I have combined Sections 2.1 “Data Sources” and 2.2 “Analytical Methods” from the original manuscript into a single section.

2.Sequential figure-by-figure narration was deleted. For each map I now report only the network statistics (nodes, links, density, size of the largest component, modularity Q and mean silhouette S) and the substantive reading that follows from them.

3.Four less essential CiteSpace visualizations were removed, including keyword timeline maps and burst-detection maps (original Figures 6–9). The section now carries five figures instead of nine.

4.The discussion was also revised to adopt a more concise and objective academic tone.

5.The revised manuscript now presents two clearly differentiated research gaps instead of overlapping descriptions..

Net effect: the bibliometric material was reduced from roughly 2,880 words and 9 figures to roughly 1,600 words and 5 figures, a reduction of about 45% in text. This change also serves Reviewer 2’s Major Comment 4; see Section C.4 below.

**Corresponding text in the revised manuscript：**

"The above analysis of the knowledge structure indicates that existing research has established a relatively clear framework oriented toward capability enhancement, namely, that digital resources primarily achieve corporate sustainability by promoting innovation capabilities, driving green innovation, and improving environmental performance. However, there remains room for expansion in two areas. First, existing research primarily focuses on the technological transformation process through which digital transformation promotes green innovation, while paying insufficient attention to how digital resources create value in different industrial contexts. ... Second, while existing research addresses topics such as information disclosure and corporate governance, it has not fully elucidated how digitalization influences ESG performance by improving the external information environment."

(Section "Literature Review and Identification of Research Gaps", p. 4)

### **Comment 1.2 — The measurement of key variables needs further clarification**

**Reviewer comment:** "The Measurement of Key Variables Needs Further Clarification — The 'Digital Transformation Index' is sourced from Shanghai University of Finance and Economics. It would be helpful to briefly explain its construction method (e.g., keyword extraction logic, weighting scheme) to enhance transparency and replicability."

**Text in the original submission:** "The 'China Listed Companies Digital Transformation Index' published by the School of Business at Shanghai University of Finance and Economics is employed. This index is constructed based on textual data from listed companies' annual reports, utilising text analysis and keyword frequency statistics. It systematically covers core dimensions such as digital technology application and digital platform development, comprehensively depicting the depth and breadth of corporate digital transformation."

(Section "Independent Variable: Digital Transformation Level", p. 35)

**My response and how the manuscript was revised:**

I appreciate this helpful suggestion. The original description provided the general source and purpose of the index but lacked sufficient details regarding its construction procedure. The revised description now states the keyword extraction logic (a purpose-built dictionary of digital keywords covering artificial intelligence, big data, cloud computing, blockchain and digital-technology application, applied to annual-report text), the quantification step (text analysis plus word-frequency counting), the normalisation step, and the direction of the resulting index.

On weighting specifically: the index is a standardised aggregation of keyword-frequency counts rather than a weighted composite with author-assigned weights. Regarding weighting, I further clarified that the index is derived from standardized keyword-frequency measures rather than an author-assigned weighted composite.

**Corresponding text in the revised manuscript:**

"This paper uses the 'China Listed Companies Digital Transformation Index,' published by the School of Business at Shanghai University of Finance and Economics, to measure the level of digital transformation among enterprises. Based on textual data from listed companies' annual reports, this index identifies content related to artificial intelligence, big data, cloud computing, blockchain, and the application of digital technologies by constructing a dictionary of digital keywords. It then quantifies the degree of digital transformation using text analysis and word frequency statistics. After standardization, a company-level digital transformation intensity indicator is derived; a higher index value indicates a higher level of digital transformation. Compared to text-based indicators developed independently by researchers, this index features a relatively mature construction framework and publicly available data sources, thereby enhancing the transparency of variable measurement and the reproducibility of research results."

(Section "Variable Definitions and Measurement", item 2 "Explanatory Variable: Level of Digital Transformation", p. 27)

### **Comment 1.3 — The measurement of green innovation suffers from a “zero inflation” problem**

**Reviewer comment:** "The Measurement of Green Innovation Suffers from a 'Zero Inflation' Problem — With over 50% of firms having zero green innovation output, this could affect the robustness of the regression results. The authors should consider addressing this, for instance by employing a Zero-Inflated model or a Tobit model as a robustness check, or at least discussing this limitation."

**Text in the original submission:** "Drawing upon research by Zhang Hui et al. [40], this study employs the annual number of green invention patent applications from the CNRDS database as the core proxy indicator. The log of the green innovation patent applications is calculated by adding one to each value to mitigate skewness."

(Section 4 "Mediating Variable: Green Innovation",p. 35)

"The Sobel test revealed that the indirect effect of green innovation was marginally significant at the 10% level (z=1.92, p=0.054) with a value of 0.0033, accounting for approximately 5.2% of the total effect. This indicates that green innovation partially mediates the relationship between digital transformation and ESG performance, partially supporting Hypothesis H2."

(Section "Mediating Effect Analysis",p. 45)

**My response and how the manuscript was revised:**

I appreciate this important comment and agree that the previous treatment of green innovation required further consideration given the large proportion of zero observations. The original log(x+1) transformation helped reduce skewness but did not fully address the distributional characteristics of the green innovation variable.

To address this concern, I made the following revisions:

1.The transmission model for the technology-oriented mechanism has been re-estimated using a Tobit model for Path a (digital transformation → green innovation), treating green innovation as a left-censored variable with a large number of zero observations. Path b and the direct effect are estimated using OLS with year and industry fixed effects and firm-level clustered standard errors.

2.The revised manuscript now explicitly reports the zero-value characteristics of green innovation in the descriptive statistics and discusses the median value of zero.

3.Based on the revised estimation results, I no longer claim a significant mediation effect of green innovation. Instead, the manuscript now discusses that although digital transformation may promote green innovation, the conversion of such innovation capability into ESG outcomes is constrained by industry-specific conditions.

These revisions improve the robustness of the empirical analysis and provide a more accurate interpretation of the role of green innovation in the digital transformation–ESG relationship.

**Corresponding text in the revised manuscript:**

"To test whether the technology-enabling mechanism corresponding to green innovation holds (H2), I use the coefficient product method (a × b) to test for indirect effects. Since the green innovation variable contains a large number of zero values (left-censored), I use a Tobit model to estimate path a to avoid OLS estimation bias; the remaining equations use OLS and control for year and industry fixed effects, with standard errors clustered at the firm level."

(Section "Model Specification" → "Transmission Effect Models for the Two Mechanisms", item 1 "Technology-Enabling Mechanism: Green Innovation Transmission Model (Tobit + OLS)", p. 31)

"As two types of mechanism variables, green innovation had a mean of 0.418 and a standard deviation of 0.777, with a median of 0, indicating strong heterogeneity in the green innovation activities of energy-intensive enterprises; the external information environment had a mean of 5.362 and a standard deviation of 7.578, with the high degree of dispersion indicating significant differences in the extent of improvement in the information environment across enterprises."

(Section "Analysis of Empirical Results" → "Descriptive Statistics and Correlation Analysis", pp. 35–36)

"Regarding the technology-enabled mechanism, Path a (digital transformation → green innovation) is marginally significant at the 10% level. Although Path b (green innovation → ESG) shows a significant positive effect, the 95% confidence interval for the indirect effect includes zero, indicating that, at the level of the full sample, green innovation cannot yet be considered a stable transmission mechanism through which digital transformation influences ESG performance. These results suggest that digital transformation possesses the potential to promote green innovation, but the further translation of this potential into ESG value remains subject to industry-specific conditions."

(Section "Analysis of Empirical Results" → "Test Results for the Two Transmission Mechanisms", p. 39)

### **Comment 1.4 — The explanation for the negative mediating effect in the high-growth group is insufficient**

**Reviewer comment:** "The Explanation for the Negative Mediating Effect in the High-Growth Group is Insufficient — The negative mediating effect in the high-growth group is attributed to 'resource crowding out.' However, theoretically, it could also be due to a 'lag effect' where digital investments have not yet yielded green outcomes. A more in-depth discussion or analysis using lag structures might be beneficial."

**Text in the original submission:** "(2) Among high-growth enterprises: This indirect effect was significantly negative (effect size = -0.0076, 95% CI = [-0.014, -0.001], p = 0.006). This characteristic is an inevitable outcome of stage-specific strategic choices: during high-growth phases, core objectives centre on short-term scale expansion and market share gains. Digital resources are systematically directed towards areas yielding rapid returns, creating a temporary conflict in resource allocation with green innovation. This leads to a reversal in the direction of transmission along the mediating pathway."

(Section "Analysis of Moderated Mediation Effects", p. 47)

"Regression results (Table 6) reveal a significantly negative coefficient for the interaction term between digital transformation and growth trajectory (β=-0.014, p<0.001), strongly supporting Hypothesis H3. ... Its underlying mechanism stems from the strategic resource allocation conflict between 'scale expansion' and 'green innovation' in energy-intensive enterprises—high growth reinforces firms' preference for short-term scale gains, thereby crowding out resources that would otherwise be allocated to green innovation."

(Section "Analysis of Moderated Mediation Effects", p. 47)

**My response and how the manuscript was revised:**

I appreciate this insightful suggestion. The reviewer raises an important point that the negative indirect effect observed in the high-growth group may have alternative explanations, including resource allocation constraints and delayed realization of green outcomes following digital investment.

After reconsidering this issue together with the revised mediation analysis, I found that the interpretation of the subgroup-specific indirect effect required stronger theoretical and empirical support. Specifically, after re-estimating the technology-oriented mechanism using the Tobit model, the green innovation pathway no longer provides sufficient evidence for a stable mediation effect. Therefore, the previous moderated mediation analysis based on this pathway may overstate the role of growth heterogeneity.

Accordingly, I made the following revisions:

1.The moderated mediation model, subgroup bootstrap analysis, negative moderation hypothesis, and related interpretation of resource crowding out have been removed from the revised manuscript.

2.Growth potential (Tobin’s Q) is retained as a control variable in the empirical specifications.

3.The theoretical framework is revised to emphasize an industry-level structural mechanism, namely asset rigidity, which reflects the constraints arising from capital intensity, asset specificity, and long equipment replacement cycles in energy-intensive industries.

4.The reviewer’s suggested lag explanation is incorporated into the revised discussion. Specifically, the delayed conversion of digital resources into green outcomes is discussed as an implication of asset rigidity, because production-system transformation and equipment upgrading require substantial time and capital commitment.

The revised manuscript therefore focuses on comparing the relative stability of two value-conversion pathways rather than explaining subgroup differences in the sign of indirect effects.

**Corresponding text in the revised manuscript:**

"For energy-intensive firms, the further embedding of digital resources into production systems is often constrained by industry-specific structural characteristics. Capital intensity, high asset specificity, and long equipment replacement cycles increase the adjustment costs associated with corporate physical assets. This study conceptualizes these structural constraints as 'asset rigidity,' referring to resource conversion constraints arising from capital commitment, asset specificity, and lengthy replacement cycles. Specifically, asset rigidity is reflected in potential barriers to transforming digital resources into green innovation outcomes. For example, in capital-intensive industries such as steel and cement, production equipment typically has long replacement cycles, and green technology upgrading requires sustained and substantial capital investment. As a result, the transformation of digital resources into green innovation outcomes may be hindered by asset lock-in effects."

(Section "Introduction", p. 7)

"Furthermore, the difference between the two is not one of degree but of conversion logic: the value of the former must be realized through the transformation of physical assets [21], while the latter is realized with relatively limited dependence on physical asset adjustments—this distinction determines that industry asset rigidity will impose asymmetric constraints on the two pathways, and it is precisely the starting point for all theoretical deductions and empirical designs in this paper."

(Section "Introduction", p. 8)

"H2: Green innovation is a key value-transformation pathway through which digital transformation influences the ESG performance of energy-intensive enterprises, but its effectiveness is constrained by the characteristics of assets in energy-intensive industries."

(Section "Research Hypotheses" → "Green Innovation as an Asset-Embedded Path", p. 24)

"H4: In the context of energy-intensive industries, the two transmission mechanisms through which digital transformation affects ESG performance exhibit different levels of value-conversion stability, with the information-embedded pathway being more stable than the asset-embedded pathway."

(Section "Research Hypotheses" → "Differential Effects of Two Value Embedding Paths", p. 26)

"Further analysis reveals that the asset-embedded pathway corresponding to green innovation has not established a stable transmission mechanism. Constrained by structural characteristics such as capital intensity and high asset specificity, the efficiency of converting digital resources into green innovation is wakened by asset rigidity, making it difficult to effectively unlock the value of technological empowerment."

(Section "Research Conclusions and Outlook" → "Key Research Conclusions", p. 47)

### **Comment 1.5 — The policy implications are somewhat generic and could be more targeted**

**Reviewer comment:** "The Policy Implications are Somewhat Generic and Could be More Targeted — The policy recommendations are broad. It is suggested to make them more specific based on the empirical results. For example, propose green innovation subsidies for low-growth firms and stricter ESG disclosure requirements for high-growth firms."

**Text in the original submission:** "To steer enterprises toward proactive transformation, governments should establish precise and effective fiscal and financial incentive systems. This includes offering targeted subsidies and tax incentives for digital and green transition projects undertaken by energy-intensive enterprises, with priority support directed toward key areas such as green technology R&D and digital infrastructure upgrades [45]. A dedicated green innovation fund should be established to reward firms that achieve significant emission reductions or demonstrate outstanding green patent output."

(Section "Government Level: Differentiated Institutional Provision and Policy Support",p. 59)

**My response and how the manuscript was revised:**

I appreciate this valuable suggestion and agree that the original policy implications were not sufficiently differentiated according to the empirical findings.

Since the growth-based moderation effect was removed after the revised analysis, growth can no longer serve as the basis for policy differentiation. Instead, I incorporated the reviewer’s suggestion by grounding the revised policy recommendations in the mechanisms supported by the empirical results, namely asset rigidity and information-governance capacity.

The following revisions were made:

1.At the firm level, the revised manuscript distinguishes between enterprises with different asset characteristics. Firms with highly specialised assets and long retrofitting cycles may prioritise the information-governance pathway, including energy monitoring, environmental data collection, and ESG disclosure capabilities, while firms with greater asset-renewal capacity may integrate digital technologies with green innovation and production upgrading.

2.At the industrial policy level, the revised discussion proposes targeted support for industries with high asset rigidity, such as steel, cement, and coking. Rather than adopting a uniform digital transformation assessment framework, policy support should focus on reducing adjustment costs through financing facilitation, tax incentives, and support for digital green technological upgrading.

3.At the ESG regulatory level, the revised manuscript proposes a tiered disclosure mechanism aligned with firms’ information-governance foundations. Enterprises with stronger digital foundations may gradually adopt more verifiable digital ESG disclosure practices, while firms with weaker information foundations may benefit from standardised disclosure templates and lightweight energy-and-carbon management tools.

In addition, several broad recommendations without direct empirical support were removed to ensure that the policy implications more closely follow from the findings of this study.

**Corresponding text in the revised manuscript:**

"At the corporate level, energy-intensive enterprises should select a digital ESG transformation path suited to the inflexible nature of their assets. For traditional manufacturing enterprises with highly specialized assets and long retrofitting cycles for existing equipment, the short-term priority should be to leverage digital technologies to enhance energy consumption monitoring, environmental data collection, and ESG disclosure capabilities. By empowering governance, they can unlock the value of digitalization and avoid blindly pursuing high-cost technological upgrades when conditions are not yet ripe. For enterprises with significant room for asset renewal and the conditions for production capacity upgrades, digital technologies can be further integrated into green R&D and production processes in conjunction with equipment upgrades and process optimization."

(Section "Policy Implications", p 48)

"At the industrial policy level, support mechanisms for green innovation should be refined to address the transformation constraints faced by industries with high asset rigidity. For industries such as steel, cement, and coking—where equipment retrofitting costs are high—policy support should not adopt a one-size-fits-all digital assessment model. Instead, it should reduce the costs of adjusting physical assets through targeted support for digital green technological upgrades, financing facilitation, and tax incentives, thereby increasing the feasibility of converting digital resources into green innovation."

(Section "Policy Implications", p. 48)

"At the ESG regulatory level, a tiered disclosure mechanism aligned with enterprises' information governance foundations should be further refined. For enterprises with strong digital foundations and favorable external information environments, verifiable digital ESG disclosure requirements should be implemented first, mandating the integration of operational condition monitoring and emissions network data to amplify the effect of governance constraints. For small and medium-sized enterprises (SMEs) with weak information foundations, support should be provided through industry-standardized disclosure templates and lightweight energy-and-carbon management SAAS tools."

(Section "Policy Implications", p. 49)

### **Comment 1.6 — Figure and table numbering is inconsistent, with citation errors**

**Reviewer comment:** "Figure and Table Numbering is Inconsistent, with Citation Errors — The manuscript contains multiple instances of 'Error! Reference source not found.' throughout the text. A thorough proofread to correct all figure/table cross-references is necessary."

**My response:**

I appreciate the reviewer’s careful observation. I apologize for the citation errors. These issues resulted from compatibility differences between word-processing software when handling automatic cross-reference fields. During the preparation of the manuscript, I used WPS Office and did not fully verify whether the figure and table references were displayed correctly after conversion to Microsoft Word format, which led to unresolved cross-reference fields appearing in the submitted file.

To address this issue, I have thoroughly checked the manuscript in Microsoft Word and corrected all figure and table references. The following revisions have been made:

1.All automatic cross-reference fields for figures and tables were checked and converted into stable text citations to prevent compatibility-related display errors.

2.All figures and tables were renumbered according to their order of appearance, and each figure and table is now cited consistently in the main text.

3.The entire manuscript was proofread in Microsoft Word, and no instance of “Error! Reference source not found.” remains in the revised clean draft.

4.Following the removal of several unnecessary CiteSpace visualizations and the addition of one marginal-effects figure, the figure set was renumbered consistently.

These revisions ensure that all figure and table citations display correctly across software environments and improve the readability and reliability of the manuscript.

### **Comment 1.7 — The language is occasionally redundant and could benefit from polishing**

**Reviewer comment:** "The Language is Occasionally Redundant and Could Benefit from Polishing — Some sections, particularly in the theory-building part, contain repetitive phrasing. Streamlining the language would improve readability and conciseness."

**My response:**

I appreciate this helpful suggestion. This issue also overlaps with Reviewer 2’s Minor Comment 3, and both comments were addressed through a comprehensive revision of the manuscript language, structure, and narrative logic.

The following revisions were made:

1.The theoretical framework was refined by reorganizing and streamlining the existing theoretical discussions. The original manuscript had already incorporated the resource-based view, dynamic capability theory, and stakeholder theory; however, some theoretical explanations and their connections with the proposed mechanisms were repetitive. In the revised manuscript, these theories are retained as the foundation of the research framework, while redundant elaborations are removed and each theoretical perspective is linked more directly to the corresponding mechanism.

2.Following the refinement of the theoretical foundation, the hypothesis development section was substantially streamlined. The revised manuscript reduces repeated theoretical reasoning and focuses each hypothesis on its core theoretical rationale and mechanism. This adjustment improves the logical connection between theoretical arguments, hypotheses, and empirical tests.

3.Beyond sentence-level editing, the overall narrative structure of the manuscript was revised. In particular, the Introduction and Literature Review sections were reorganized to improve the logical progression from research background, existing knowledge, research gaps, and theoretical motivation. Redundant explanations and repeated statements of similar research motivations were consolidated to ensure that each section serves a distinct purpose.

4.The empirical analysis section was also refined. The revised manuscript focuses on the central analyses and key empirical evidence directly related to the research questions, while removing excessive descriptions and interpretations of secondary results. This revision improves conciseness while maintaining the completeness and rigor of the empirical discussion.

5.Overstated rhetorical expressions and unnecessary evaluative wording were revised throughout the manuscript to improve academic objectivity and precision. Claims and descriptions were adjusted to better match the strength of the statistical evidence and avoid excessive interpretation.

These revisions improve the clarity, conciseness, and readability of the manuscript while ensuring that the theoretical arguments and empirical findings are presented in a more balanced and precise manner.

# **3. Response to Reviewer 2 — Major comments**

### **Major 1 — OLS applied to a zero-inflated, right-skew mediator**

**Reviewer comment:** "The authors measure the mediating variable, 'Green Innovation,' as the natural logarithm of the number of green invention patent applications plus one. The descriptive statistics reveal that the median is 0 ... Applying standard Ordinary Least Squares (OLS) regressions to count data with a heavy right skew and zero-inflation violates fundamental statistical assumptions (normality and homoscedasticity of residuals), leading to biased and inconsistent estimates. — Need to address: The authors must re-estimate the models predicting green innovation using models appropriate for count or censored data, such as a Poisson regression, Negative Binomial regression, or a Tobit model."

**Text in the original submission:** "Drawing upon research by Zhang Hui et al. [40], this study employs the annual number of green invention patent applications from the CNRDS database as the core proxy indicator. The log of the green innovation patent applications is calculated by adding one to each value to mitigate skewness."

(Section "Mediating Variable: Green Innovation", p. 35)

"To examine the mediating role of green innovation in the relationship between digital transformation and ESG performance in energy-intensive enterprises (H2), the following models Ire constructed based on the three-step approach proposed by Baron and Kenny (1986): Step 1 (Total Effect Model) ... Step 2 (Path a Model) ... Step 3 (Direct Effect and Path b Model) ... The mediation effect (indirect effect) is a×b, and the total effect is c=c'+(a×b). If both a and b are statistically significant and the Sobel test is significant, partial mediation is supported."

(Section"Mediation Effect Model", pp. 36–37)

**My response and how the manuscript was revised:**

I appreciate this important methodological concern and agree that the original estimation approach did not sufficiently account for the distributional characteristics of the green innovation variable. The mediation analysis has therefore been re-estimated using models more appropriate for the observed data structure, which led to a more cautious interpretation of the green innovation pathway.

1.Green innovation (with substantial zero observations and a median value of 0). Path a is now estimated using a Tobit model with left-censoring at zero, while path b and the direct effect are estimated using OLS with year and industry fixed effects and firm-clustered standard errors. The revised results show that path a is only marginally significant (a = 0.0189, SE = 0.0097, p = 0.053). The indirect effect (a×b) is 0.0143 (SE = 0.0085, p = 0.094), with the 95% confidence interval containing zero. Therefore, the revised manuscript no longer treats green innovation as a stable mediating mechanism.

2.External information environment (count variable with over-dispersion). As an additional governance-oriented mechanism introduced in the revised framework, the external information environment is estimated using negative binomial regression for path a, given that its variance substantially exceeds its mean. The average marginal effect is reported to facilitate interpretation. The results show a significant indirect effect (0.044, SE = 0.011, p < 0.001, 95% CI [0.022, 0.066]), accounting for 32.6% of the total effect.

3.The mediation analysis has been revised accordingly. Table 5 now reports the estimation method for each pathway, including the total effect, path a, path b, direct effect, indirect effect, standard errors, p-values, confidence intervals, and mediation proportion. The descriptive statistics section also explicitly reports the distributional characteristics of both mechanism variables.

Regarding the choice of estimator, a zero-inflated model was not adopted because such models assume two separate data-generating processes: one determining whether a firm produces innovation output and another determining the level of output conditional on innovation. Given the absence of a clear basis for distinguishing these two processes in the current sample, the Tobit specification was adopted as a more appropriate approach for addressing the large number of zero observations.

**Corresponding text in the revised manuscript:**

"To test whether the technology-enabling mechanism corresponding to green innovation holds (H2), I use the coefficient product method (a × b) to test for indirect effects. Since the green innovation variable contains a large number of zero values (left-censored), I use a Tobit model to estimate path a to avoid OLS estimation bias; the remaining equations use OLS and control for year and industry fixed effects, with standard errors clustered at the firm level."

(Section "Model Specification" → "Transmission Effect Models for the Two Mechanisms", item 1 "Technology-Enabling Mechanism: Green Innovation Transmission Model (Tobit + OLS)", p. 31)

"To test whether the governance empowerment mechanism associated with the external information environment holds (H3)—given that the external information environment is a count variable with highly discrete characteristics—a negative binomial regression model is employed; ESG is treated as a continuous variable, and the path b and direct effects are estimated using ordinary least squares (OLS). ... Due to the nonlinear nature of the model, I compute the average marginal effect (AME)..."

(Section "Transmission Effect Models for the Two Mechanisms", item 2 "Governance Empowerment Mechanism: External Information Environment Transmission Model (Negative Binomial Regression + OLS)", pp. 32-33）

"As two types of mechanism variables, green innovation had a mean of 0.418 and a standard deviation of 0.777, with a median of 0, indicating strong heterogeneity in the green innovation activities of energy-intensive enterprises; the external information environment had a mean of 5.362 and a standard deviation of 7.578, with the high degree of dispersion indicating significant differences in the extent of improvement in the information environment across enterprises."

(Section "Analysis of Empirical Results" → "Descriptive Statistics and Correlation Analysis", p. 35)

"Regarding the technology-enabled mechanism, Path a (digital transformation → green innovation) is marginally significant at the 10% level. Although Path b (green innovation → ESG) shows a significant positive effect, the 95% confidence interval for the indirect effect includes zero, indicating that, at the level of the full sample, green innovation cannot yet be considered a stable transmission mechanism through which digital transformation influences ESG performance. These results suggest that digital transformation possesses the potential to promote green innovation, but the further translation of this potential into ESG value remains subject to industry-specific conditions."

(Section "Analysis of Empirical Results" → "Test Results for the Two Transmission Mechanisms", p. 39)

"In contrast, the governance-empowerment pathway exhibits more stable statistical characteristics. Digital transformation significantly improves a firm's external information environment and further promotes enhanced ESG performance, with the mediating effect accounting for 32.6% of the total effect."

(Section "Test Results for the Two Transmission Mechanisms", p. 39)

### **Major 2 — A one-year lag is insufficient to address endogeneity**

**reviewer comment:** "In corporate finance and management research, a simple one-year lag is insufficient to resolve endogeneity concerns. Firms with stronger ESG performance often have better access to financing, allowing them to invest more heavily in digital transformation (simultaneity/reverse causality). Furthermore, the risk of omitted variable bias remains high. — Need to address: The authors must implement a more robust identification strategy to address endogeneity. Acceptable approaches include Instrumental Variables (IV/2SLS), Generalized Method of Moments (GMM), or Propensity Score Matching with Difference-in-Differences (PSM-DID). Without these robustness checks, the paper can only claim correlations, not causal impacts."

**My response and how the manuscript was revised:**

I appreciate this important concern. I agree that a one-period lag alone cannot fully address potential endogeneity arising from reverse causality and omitted-variable bias. In the original manuscript, the lag structure and additional robustness analyses provided limited evidence regarding endogeneity beyond temporal ordering. Accordingly, I have incorporated additional analyses to further examine the robustness of the relationship between digital transformation and ESG performance.

First, an instrumental variable (IV) approach based on two-stage least squares (2SLS) has been introduced. Following prior studies, the contemporaneous average digital transformation level of other firms within the same industry and year (IV_IndustryMean) is used as the instrumental variable. This instrument captures the influence of industry-level digital diffusion on firms’ digital transformation decisions while being less directly related to firm-specific ESG outcomes. The first-stage results show that the coefficient of the instrument is 0.377 and significant at the 1% level. The Kleibergen–Paap rk LM statistic is 17.98 (p < 0.01), rejecting the under-identification concern. The first-stage F-statistic is 18.80, exceeding the Stock–Yogo 10% critical value of 16.38, suggesting that weak-instrument concerns are unlikely to be a major issue.

The second-stage results show that the coefficient of digital transformation remains positive but does not reach conventional statistical significance. This result is consistent with the revised interpretation of the manuscript: digital transformation is positively associated with ESG performance, but its direct association is not robust across specifications. Instead, the ESG value of digital transformation is more likely to be realized through specific value-conversion mechanisms.

Second, a firm fixed-effects specification has been added to further control for unobserved time-invariant firm characteristics. This additional test reduces concerns that persistent differences among firms may drive the observed relationship between digital transformation and ESG performance.

Third, the wording of the manuscript has been revised throughout. Following the reviewer’s suggestion, causal expressions such as “impact” and “effect” have been replaced with more appropriate terms such as “positive association,” “relationship,” and “linkage” where strict causal identification is not established. Hypothesis H1 has also been revised accordingly.

Regarding alternative approaches, dynamic panel GMM and PSM-DID were not adopted in the revised manuscript. Dynamic GMM is more suitable for settings involving dynamic dependent variables and appropriate internal instruments, while the current research design does not provide sufficiently strong conditions for this approach. PSM-DID generally requires a clearly defined exogenous treatment event and comparable treated and control groups; however, digital transformation in this study is measured as a continuous intensity index rather than a discrete adoption event. Nevertheless, the revised manuscript acknowledges that future research based on quasi-natural experiments, such as digital transformation pilot programmes or ESG disclosure policies, may provide stronger causal identification.

**Corresponding text in the revised manuscript:**

"To alleviate potential endogeneity issues such as reverse causality and omitted variable bias between the one-period lagged digital transformation and ESG performance, I employ the instrumental variable approach, namely two-stage least squares (2SLS) estimation. Following existing studies (e.g., Fisman & Svensson [43]), I adopt the average value of DT_Index for other firms within the same industry and year as the instrumental variable (IV_IndustryMean). The relevance of this instrument is based on the peer effect of digital transformation within the same industry, as firms' digitalization processes may influence the digital transformation decisions of their industry peers. Considering that industry-level common factors may simultaneously affect firms' ESG performance, this study further controls for industry and year fixed effects to mitigate the influence of industry-wide shocks. The instrumental variable results are therefore treated as supplementary evidence to address potential endogeneity concerns."

(Section "Robustness Test Models", item 3 "Addressing Endogeneity", pp. 34-35)

"The results of first stage show that the instrument coefficient is 0.377, which is significantly positive at the 1% level ... The Kleibergen-Paap rk LM statistic is 17.98 and is significant at the 1% level; the F-statistic for Stage 1 is 18.80, which is higher than the Stock-Yogo 10% critical value of 16.38, indicating that there is no significant weak instrument problem. The results of the second stage show that the coefficient for digital transformation remains positive but does not reach statistical significance."

(Section "Robustness Tests", item 3 "Treatment of Endogeneity", p. 44)

"After controlling for year fixed effects, industry fixed effects, and firm-level control variables, the coefficient for the digital transformation index (one period lagged) is 0.0467 (t = 1.50); the direction is positive but does not pass the conventional significance test ... These two sets of results indicate that digital transformation is generally positively associated with ESG performance, but this association weakens significantly when firm-level characteristics are included."

(Section "Analysis of Empirical Results" → "Benchmark Regression Results", p. 37)

### **Major 3 — Baron–Kenny and the Sobel test should be replaced**

**reviewer comment:** "The manuscript relies on the Baron and Kenny (1986) causal steps approach and the Sobel test to establish mediation. The Baron and Kenny method is widely considered outdated in modern econometrics and psychology, as it suffers from low statistical power. Similarly, the Sobel test assumes a normal distribution for the indirect effect, which is rarely the case. — Need to address: The authors should use a Bootstrapping approach (e.g., using the PROCESS macro or Structural Equation Modeling) to test the significance of the indirect effect for the entire mediation model, replacing the Baron and Kenny framework entirely."

**Text in the original submission:** "To examine the mediating role of green innovation in the relationship between digital transformation and ESG performance in energy-intensive enterprises (H2), the following models Ire constructed based on the three-step approach proposed by Baron and Kenny (1986): Step 1 (Total Effect Model) ... Step 2 (Path a Model) ... Step 3 (Direct Effect and Path b Model) ... The mediation effect (indirect effect) is a×b, and the total effect is c=c'+(a×b). If both a and b are statistically significant and the Sobel test is significant, partial mediation is supported."

(Section "Mediation Effect Model", p. 38)

"The Sobel test revealed that the indirect effect of green innovation was marginally significant at the 10% level (z=1.92, p=0.054) with a value of 0.0033, accounting for approximately 5.2% of the total effect. This indicates that green innovation partially mediates the relationship between digital transformation and ESG performance, partially supporting Hypothesis H2."

(Section "Mediating Effect Analysis", p. 45)

**My response and how the manuscript was revised:**

I appreciate the reviewer’s valuable suggestion regarding the limitations of the Baron–Kenny causal steps approach and the Sobel test. Following this suggestion, I have further revised the mediation analysis by removing the causal-steps decision framework and adding a bootstrap-based robustness examination of the indirect effects.

Specifically, the revised manuscript no longer determines mediation based on the sequential significance of individual paths. Instead, inference is based on the magnitude and statistical significance of the indirect effect itself. The revised analysis reports the indirect effect together with its standard error, confidence interval, and statistical significance.

Because the two mediators in this study have different distributional characteristics, I continue to employ estimators appropriate for each mediation path. Specifically, the green innovation equation is estimated using a Tobit model due to the left-censored nature of the variable, while the external information environment equation is estimated using a negative binomial model with average marginal effects. The outcome equations are estimated using OLS with firm-clustered standard errors.

To directly address the reviewer’s concern regarding the distributional assumption of the Sobel test, I additionally conduct a firm-level bootstrap test of the indirect effects. The bootstrap procedure repeatedly resamples firms with replacement and re-estimates the complete mediation process. The results are reported in the newly added Table X.

The bootstrap results confirm the original conclusions. The indirect effect of green innovation remains statistically insignificant (Indirect effect = 0.01334, 95% CI = [-0.00456, 0.03123]), indicating that the technology-enabled pathway does not constitute a stable transmission mechanism in the full sample. In contrast, the indirect effect through the external information environment remains significantly positive (Indirect effect = 0.00173, 95% CI = [0.00083, 0.00262]), supporting the robustness of the governance-enabled pathway.

For the bootstrap estimation of the information environment mechanism, I use ln(Transparency) rather than the raw Transparency measure. This adjustment is made for two reasons. First, the original Transparency measure exhibits substantial right-skewness, and logarithmic transformation improves distributional properties and reduces the influence of extreme observations. Second, during bootstrap resampling, the negative binomial estimation based on the raw Transparency measure occasionally encounters convergence problems due to the sparse distribution of mediator values. The logarithmic transformation improves estimation stability across bootstrap replications. Importantly, this transformation does not change the substantive conclusion: the indirect effect remains positive and statistically significant.

Accordingly, I have added the bootstrap robustness test and revised the discussion of mediation effects in Section 4.4.3.

**Corresponding text in the revised manuscript:**

"To test whether the technology-enabling mechanism corresponding to green innovation holds (H2), I use the coefficient product method (a × b) to test for indirect effects. Since the green innovation variable contains a large number of zero values (left-censored), I use a Tobit model to estimate path a to avoid OLS estimation bias; the remaining equations use OLS and control for year and industry fixed effects, with standard errors clustered at the firm level."

(Section "Model Specification" → "Transmission Effect Models for the Two Mechanisms", item 1 "Technology-Enabling Mechanism: Green Innovation Transmission Model (Tobit + OLS)", p. 31)

"Notes: For Green_Innovation, Path (a) is estimated using a Tobit model (left-censored at 0), while Path (b) and the direct effect are estimated via OLS. Indirect effects are computed with the Delta method (Sobel test). For Transparency, Path (a) is estimated via negative binomial regression, and average marginal effects (AME) are calculated. Path (b) and the direct effect are estimated using OLS, and the indirect effect is derived based on the Delta method."

(Note to Table 5 "Mediation Effect Estimation Results", p. 40)

"Notes: This table reports the bootstrap robustness test of the indirect effects. The bootstrap procedure resamples firms with replacement and re-estimates the mediation models. The procedure is based on 5,000 replications. For the Transparency mechanism, ln(Transparency) is used as the mediator in the bootstrap estimation to improve distributional properties and estimation stability during resampling. Confidence intervals are based on bootstrap standard errors."

(Note to Table 6 "Mediation Effect Estimation Results", p. 41)

"Regarding the technology-enabled mechanism, Path a (digital transformation → green innovation) is marginally significant at the 10% level. Although Path b (green innovation → ESG) shows a significant positive effect, the 95% confidence interval for the indirect effect includes zero, indicating that, at the level of the full sample, green innovation cannot yet be considered a stable transmission mechanism through which digital transformation influences ESG performance. These results suggest that digital transformation possesses the potential to promote green innovation, but the further translation of this potential into ESG value remains subject to industry-specific conditions."

(Section "Analysis of Empirical Results" → "Test Results for the Two Transmission Mechanisms", p. 40)

### **Major 4 — The bibliometric section is disproportionate and creates structural imbalance**

**reviewer comment:** "A disproportionate amount of the manuscript is dedicated to a CiteSpace bibliometric analysis simply to justify the choice of variables and identify the research gap. This creates a structural imbalance, making the manuscript read like two separate, disjointed papers ... — Need to address: The bibliometric section should be drastically condensed (to 2-3 pages maximum) or moved entirely to the Supplementary Materials/Appendix. The core focus of the manuscript must be the econometric model, its robustness, and the economic interpretation of the findings."

**My response and how the manuscript was revised:**

I appreciate this important observation. I agree that the original manuscript devoted excessive space to the bibliometric analysis, which weakened the continuity between the literature review and the empirical investigation and made the manuscript appear as two separate studies. Following the reviewer’s suggestion, I substantially condensed and repositioned the bibliometric analysis so that it now serves only to contextualize the research gap rather than constitute an independent analytical component.

Specifically, I made the following revisions:

1.The bibliometric section was substantially shortened by removing redundant descriptive analyses, including keyword evolution and burst-detection results. The remaining analysis focuses only on the evidence necessary to identify the research gaps related to the technological pathway and the information-based governance pathway.

2.The original six-subsection bibliometric analysis was reorganized into a concise section entitled **“Literature Review and the Focus of This Study.”** The revised section integrates bibliometric evidence with prior theoretical studies and no longer presents CiteSpace results as independent findings.

3.The manuscript has been structurally rebalanced. The empirical sections, including variable construction, model specification, robustness analyses, and mechanism tests, now constitute the central contribution of the study, while the literature review serves only as background support.

I retained a concise bibliometric discussion in the main text because it provides complementary evidence for identifying the underexplored governance-related mechanism in existing research. However, I have clarified that the theoretical framework and mechanism development are grounded in theoretical reasoning and prior literature, with bibliometric findings serving only as supplementary evidence.

**Corresponding text in the revised manuscript:**

"The above analysis of the knowledge structure indicates that existing research has established a relatively clear framework oriented toward capability enhancement, namely, that digital resources primarily achieve corporate sustainability by promoting innovation capabilities, driving green innovation, and improving environmental performance. however, there remains room for expansion in two areas. First, existing research primarily focuses on the technological transformation process through which digital transformation promotes green innovation, while paying insufficient attention to how digital resources create value in different industrial contexts. ... Second, while existing research addresses topics such as information disclosure and corporate governance, it has not fully elucidated how digitalization influences ESG performance by improving the external information environment."

(Section "Literature Review and Identification of Research Gaps" → "Literature Review and the Focus of This Study", p. 15)

# **4. Response to reviewer 2 — Minor comments**

### **Minor 1 — Open data: the compiled dataset and replication code should be uploaded**

**reviewer comment:** "The authors state that the raw data Ire obtained from third-party databases (CSMAR and CNRDS) ... PLOS ONE has a strict open-data policy. — Need to address: To ensure perfect replicability without violating third-party copyright of the raw databases, the authors should upload their final, compiled, and cleaned dataset (e.g., in .csv or .dta format) along with the replication code (Stata do-files or R scripts) as Supporting Information."

**My response:**

I appreciate the reviewer’s important suggestion regarding data availability and research reproducibility. I fully recognize the importance of transparency in empirical research and carefully considered the possibility of making the dataset publicly available.

However, the complete compiled dataset used in this study cannot be uploaded as Supporting Information because the underlying firm-level data were obtained from third-party commercial databases, including CSMAR and CNRDS. These databases are subject to specific licensing agreements and usage restrictions, which do not permit researchers to redistribute the original database records or compiled datasets containing information derived from these proprietary sources. Therefore, publicly releasing the complete dataset would conflict with the terms of use established by the data providers.

To facilitate reproducibility within these constraints, I can provide the replication code upon request, including the procedures for sample selection, data processing, variable construction, and empirical estimation. In addition, I have clarified the data sources, sample construction process, and variable measurement procedures in the revised manuscript, so that researchers with legitimate access to the same databases can reproduce the empirical analysis.

For information manually collected from publicly available annual reports, the relevant collection procedures and processing rules can also be documented if needed.

Therefore, although the proprietary nature of the third-party databases prevents the public release of the compiled dataset, I have made every effort to enhance transparency while complying with the legal and contractual restrictions associated with the underlying data sources.

### **Minor 2 — Figure resolution, overlapping labels and mixed Chinese/English characters**

**reviewer comment:** "Several figures generated via CiteSpace suffer from poor resolution, overlapping text, and a confusing mix of English and Chinese characters. — Need to address: All figures must be redesigned or exported at a higher resolution (minimum 300 dpi) to ensure all node labels are legible and clearly translated into English, in accordance with the journal's formatting guidelines."

**My response:**

I appreciate the reviewer’s careful observation regarding the quality and readability of the figures. I agree that the original CiteSpace figures did not fully meet the journal’s requirements in terms of resolution, label clarity, and language consistency.

I have revised the figure set substantially rather than simply rescaling the original images, as enlarging the previous raster-based figures would not have improved the readability of the node labels.

Specifically:

1.Four figures containing the most severe label congestion, including the timeline maps and burst-detection maps, were removed to improve the overall readability and reduce unnecessary visual complexity.

2.The retained figures were regenerated from CiteSpace at high resolution and prepared according to the journal’s figure formatting requirements. The revised figures are provided as separate figure files rather than embedded low-resolution images within the manuscript.

3.All Chinese keyword labels in the CNKI-based maps were translated into English to ensure consistency across figures. Font sizes were increased, while node-label density was reduced by removing low-frequency and peripheral nodes where necessary. Overlapping labels were further addressed through layout adjustment and selective node presentation.

4.Figure captions were rewritten to improve self-contained interpretation by specifying the data source, retrieval period, and relevant network parameters.

These revisions have substantially improved the visual clarity and consistency of the CiteSpace figures in the revised manuscript.

### **Minor 3 — Hyperbolic language should be replaced with an objective, neutral tone**

**reviewer comment:** "The manuscript occasionally uses hyperbolic language (e.g., 'unprecedented,' 'exponential growth,' 'profoundly reflects'). — Need to address: Please review the manuscript to ensure a more objective, neutral, and scientifically rigorous tone throughout the text. Let the data and the statistical significance speak for the magnitude of the findings."

**Text in the original submission:** "It has directly driven the establishment of the 'dual carbon' goals, turning them into an unprecedented, extensive, and profound systemic transformation of the economy and society."

(Section "Introduction", p. 1)

"The year 2022 marked a pivotal turning point for research in this field within the Chinese context, with Chinese publications demonstrating an exponential growth trend. The volume of publications from 2022 to 2025 should a dramatic surge compared to the cumulative total of the previous 15 years."

(Section "Evolution of the Field", p. 10)

"Regression results (Table 6) reveal a significantly negative coefficient for the interaction term between digital transformation and growth trajectory (β=-0.014, p<0.001), strongly supporting Hypothesis H3. ... Its underlying mechanism stems from the strategic resource allocation conflict between 'scale expansion' and 'green innovation' in energy-intensive enterprises—high growth reinforces firms' preference for short-term scale gains, thereby crowding out resources that would otherwise be allocated to green innovation."

(Section "Analysis of Moderated Mediation Effects", p. 47)

**My response and how the manuscript was revised:**

I appreciate the reviewer’s suggestion regarding the tone and wording of the manuscript. I agree that some expressions in the original version were overly strong and could imply a level of certainty beyond the evidence provided. I have therefore conducted a systematic revision of the manuscript to ensure a more objective and evidence-based presentation.

Specifically:

1.The expressions highlighted by the reviewer, including “unprecedented,” “exponential growth,” and “profoundly reflects,” have been removed or replaced with more neutral descriptions. Similar formulations that may imply excessive certainty have also been revised where appropriate.

2.Descriptions of publication trends are now presented in a more objective manner. For example, the previous characterization of research growth as “exponential” has been replaced with descriptions such as “an increasing trend” based on the observed publication pattern.

3.Following the reviewer’s suggestion, the revised manuscript relies more explicitly on statistical evidence to determine the strength of conclusions. Insignificant coefficients are now described as showing no significant association, marginally significant results are identified as such, and potentially overstated interpretations of nonlinear relationships have been avoided.

4.Interpretations of mechanisms and variables have also been refined. For example, analyst coverage is now described as an observable proxy for the external information environment rather than a direct measure of information transparency, and the limitations of this proxy are discussed in the revised manuscript.

Through these revisions, I have aimed to ensure that the manuscript presents its findings with appropriate caution and allows empirical evidence to determine the magnitude and implications of the results..

**Corresponding text in the revised manuscript:**

"Against the backdrop of China's green transition, 'promoting green and low-carbon economic and social development has become a key requirement for achieving high-quality development' [1]. This policy orientation highlights the central role of green development in China's pursuit of modernization and has elevated the 'dual carbon' goals into a comprehensive transformation of the economic and social system."

(Section "Introduction", p. 2)

"It should be noted that, since the squared term reached only marginal significance and the marginal effects plot reflects trends within the sample range, this study does not interpret these findings as a strict U-shaped relationship or a clear threshold effect, but rather regards them as supporting evidence for the gradual release of digital value."

(Section "Robustness Tests", item 2 "Nonlinear (Squared Term) Test", p. 43)

"It should be noted that analyst coverage is not information transparency itself, but rather an observable proxy for improvements in a company's external information environment. ... Given that this indicator may still be influenced by factors such as firm size and market attention, this paper controls for relevant factors in the model and further discusses its limitations in Section 5.4."

(Section "Variable Definitions and Measurement", item 4 "Mediating Variables", pp. 28–29)

"First, in terms of variable measurement, this paper still has certain limitations. Constrained by data availability, this paper uses analyst coverage and patent counts to measure the external information environment and green innovation level, respectively. Although these proxies can, to some extent, characterize the relevant mechanisms, they still cannot fully capture the dynamic changes in firms' information governance capabilities and innovation quality."

(Section "Research Limitations and Prospects", p. 50)

# **5. Summary of principal changes to the manuscript**

1.The title, abstract, and keywords were revised to better reflect the central perspective of the study, namely that the ESG value of digital transformation depends on different modes of value embedding.

2.The bibliometric analysis was substantially condensed from approximately 2,880 words and 9 figures to approximately 1,600 words and 5 figures. It was reorganized into a concise literature-review section and repositioned to support the identification of research gaps rather than function as an independent analytical component (R1.1, R2 Major 4).

3.The theoretical framework was reconstructed around a unified logic of **resource-based view → value conversion → ESG performance**. Dynamic capability theory and stakeholder theory were integrated to explain the two transmission mechanisms, while the redundant “Bibliometric Support” subsection was removed (R1.7, R2 Minor 3).

4.The concept of **asset rigidity** was introduced and clearly defined as an important structural characteristic of energy-intensive industries. It was incorporated into the theoretical framework as a contextual condition explaining differences in the effectiveness of alternative value-embedding pathways (R1.4).

5.The hypotheses were reformulated to align with the revised theoretical framework: H1 examines the relationship between digital transformation and ESG performance; H2 focuses on the asset-embedded pathway through green innovation and its contextual constraints; H3 examines the information-embedded pathway through the external information environment; and H4 investigates the differentiated transmission characteristics of the two pathways.

6.The green innovation mechanism was re-estimated using a Tobit model. Based on the updated indirect-effect evidence, the manuscript no longer claims a statistically robust mediation effect for the green innovation pathway (R1.3, R2 Major 1).

7.A second transmission mechanism, the external information environment, was introduced and measured using analyst coverage. The corresponding Path (a) estimation was conducted using negative binomial regression with average marginal effects reported, providing a more appropriate treatment for the count-based mediator (R2 Major 1).

8.The traditional Baron–Kenny causal-steps approach was no longer used as the criterion for mediation inference. Mediation conclusions are now based on indirect effects, confidence intervals, bootstrap robustness tests, and mediation proportions (R2 Major 3).

9.Additional robustness analyses were conducted, including instrumental-variable estimation using an industry-peer instrument with first-stage diagnostics, firm fixed-effects specifications, and nonlinear specifications with a centred quadratic term (R2 Major 2).

10.The moderated mediation framework and related growth-moderation interpretation were removed after reassessing their theoretical and empirical support. Firm growth potential was retained as a control variable to avoid overstating conditional mechanisms (R1.4).

11.The measurement procedure of the Digital Transformation Index was described in greater detail, including keyword dictionary construction, text analysis, word-frequency counting, standardization procedures, and indicator direction adjustments (R1.2).

12.The policy implications were rewritten to ensure that each recommendation is directly derived from the empirical findings and differentiated according to firms’ asset rigidity and information-governance conditions (R1.5).

13.All figure and table cross-references were rebuilt according to PLOS formatting requirements, and unresolved reference fields were removed (R1.6).

14.The CiteSpace figures were regenerated at high resolution with English-only labels, improved readability, and separate figure files prepared according to journal formatting requirements (R2 Minor 2).

15.The Data Availability Statement was revised to clarify the accessibility of research materials and the restrictions associated with third-party commercial databases. Replication materials can be provided subject to data-use restrictions (R2 Minor 1).

The revised manuscript provides a more focused theoretical framework, a more rigorous empirical strategy, and a more cautious interpretation of the mechanisms based on the updated evidence. I appreciate the constructive comments from the Editor and reviewers, which have substantially improved the clarity, methodological rigor, and contribution of the manuscript.

Sincerely,

Weiran Sun

School of Management, Nanjing University of Posts and Telecommunications

Nanjing 210003, Jiangsu, China

b23130508@njupt.edu.cn
