# Supplementary material for: Beyond digital investment: How value embedding modes shape ESG performance in energy-intensive firms
Source: PLoS One. 2026 Sep 21;21(9):e0343345. doi: 10.1371/journal.pone.0343345 (PMC13592625; doi:10.1371/journal.pone.0343345)
Supplement: Peer review history

# Peer review: Decision Letter 0

**DOI:** 10.1371/journal.pone.0343345.r001

**Authors:** Juan Trinidad-Segovia

6 Mar 2026

PLOS One

Dear Dr. Weiran,

Thank you for submitting your manuscript to PLOS ONE. In view of the referees’ feedback and my own reading of your paper, we invite you to address all issues noted below. We consider these issues to be major in nature, requiring more than a superficial or minor revision.

=====================================

The manuscript addresses a relevant and interesting research topic. However, the reviewers have raised several concerns regarding the robustness of the methodological approach and the econometric analysis, particularly in relation to the modelling of green innovation, potential endogeneity issues, and the mediation analysis. These issues must be carefully addressed to strengthen the validity of the results.

In addition, the manuscript would benefit from improvements in structure and clarity, including a more concise presentation of the bibliometric analysis and clarification of some variables and figures.

Since we believe that the manuscript still has potential to be considered for publication, we invite you to revise the manuscript following the reviewers’ suggestions and resubmit it for a new round of evaluation.

=====================================

Please submit your revised manuscript by Apr 20 2026 11:59PM. If you will need more time than this to complete your revisions, please reply to this message or contact the journal office at plosone@plos.org. When you're ready to submit your revision, log on to https://www.editorialmanager.com/pone/ and select the 'Submissions Needing Revision' folder to locate your manuscript file.

- A letter that responds to each point raised by the academic editor and reviewer(s). You should upload this letter as a separate file labeled 'Response to Reviewers'.
- A marked-up copy of your manuscript that highlights changes made to the original version. You should upload this as a separate file labeled 'Revised Manuscript with Track Changes'.
- An unmarked version of your revised paper without tracked changes. You should upload this as a separate file labeled 'Manuscript'.

If you would like to make changes to your financial disclosure, please include your updated statement in your cover letter. Guidelines for resubmitting your figure files are available below the reviewer comments at the end of this letter.

If applicable, we recommend that you deposit your laboratory protocols in protocols.io to enhance the reproducibility of your results. Protocols.io assigns your protocol its own identifier (DOI) so that it can be cited independently in the future. For instructions see: https://journals.plos.org/plosone/s/submission-guidelines#loc-laboratory-protocols. Additionally, PLOS ONE offers an option for publishing peer-reviewed Lab Protocol articles, which describe protocols hosted on protocols.io. Read more information on sharing protocols at https://plos.org/protocols?utm_medium=editorial-email&utm_source=authorletters&utm_campaign=protocols.

We look forward to receiving your revised manuscript.

Kind regards,

Juan E. Trinidad-Segovia, PhD

Section Editor

PLOS One

Journal Requirements:

When submitting your revision, we need you to address these additional requirements.

1. Please ensure that your manuscript meets PLOS ONE's style requirements, including those for file naming. The PLOS ONE style templates can be found at

https://journals.plos.org/plosone/s/file?id=wjVg/PLOSOne_formatting_sample_main_body.pdf and https://journals.plos.org/plosone/s/file?id=ba62/PLOSOne_formatting_sample_title_authors_affiliations.pdf

2. PLOS requires an ORCID iD for the corresponding author in Editorial Manager on papers submitted after December 6th, 2016. Please ensure that you have an ORCID iD and that it is validated in Editorial Manager. To do this, go to ‘Update my Information’ (in the upper left-hand corner of the main menu), and click on the Fetch/Validate link next to the ORCID field. This will take you to the ORCID site and allow you to create a new iD or authenticate a pre-existing iD in Editorial Manager.

3. In the online submission form, you indicated that your data is available only on request from a third party. Please note that your Data Availability Statement is currently missing [the name of the third party contact or institution / contact details for the third party, such as an email address or a link to where data requests can be made]. Please update your statement with the missing information.

4. Your abstract cannot contain citations. Please only include citations in the body text of the manuscript, and ensure that they remain in ascending numerical order on first mention.

5. Please ensure that you refer to Figure 4-10 in your text as, if accepted, production will need this reference to link the reader to the figure.

6. We note you have included a table to which you do not refer in the text of your manuscript. Please ensure that you refer to Table 1-7 in your text; if accepted, production will need this reference to link the reader to the Table.

7. Please remove your figures from within your manuscript file, leaving only the individual TIFF/EPS image files, uploaded separately. These will be automatically included in the reviewers’ PDF**.**

8. If the reviewer comments include a recommendation to cite specific previously published works, please review and evaluate these publications to determine whether they are relevant and should be cited. There is no requirement to cite these works unless the editor has indicated otherwise.

[Note: HTML markup is below. Please do not edit.]

Reviewers' comments:

Reviewer's Responses to Questions

**Comments to the Author**

1. Is the manuscript technically sound, and do the data support the conclusions?

Reviewer #1: Partly

Reviewer #2: No

**********

2. Has the statistical analysis been performed appropriately and rigorously?

Reviewer #1: No

Reviewer #2: No

**********

3. Have the authors made all data underlying the findings in their manuscript fully available?

The PLOS Data policy

Reviewer #1: No

Reviewer #2: No

**********

4. Is the manuscript presented in an intelligible fashion and written in standard English?

Reviewer #1: Yes

Reviewer #2: Yes

**********

Reviewer #1: This paper examines how energy-intensive enterprises can enhance their ESG performance through digital transformation in the context of China's "dual carbon" goals. It constructs a theoretical model that incorporates green innovation as a mediating variable and corporate growth potential as a moderating variable, using a mixed-method approach that combines bibliometric analysis with empirical testing.

The following suggestions are offered for your reference:

The Literature Review Section is Somewhat Repetitive and Could Be Condensed

The descriptions in Section 2 regarding "Knowledge Structure" and "Frontier Evolution" contain some redundancy. It is recommended to merge or streamline these parts, highlighting core findings rather than interpreting each figure sequentially.

The Measurement of Key Variables Needs Further Clarification

The "Digital Transformation Index" is sourced from Shanghai University of Finance and Economics. It would be helpful to briefly explain its construction method (e.g., keyword extraction logic, weighting scheme) to enhance transparency and replicability.

The Measurement of Green Innovation Suffers from a "Zero Inflation" Problem

With over 50% of firms having zero green innovation output, this could affect the robustness of the regression results. The authors should consider addressing this, for instance by employing a Zero-Inflated model or a Tobit model as a robustness check, or at least discussing this limitation.

The Explanation for the Negative Mediating Effect in the High-Growth Group is Insufficient

The negative mediating effect in the high-growth group is attributed to "resource crowding out." However, theoretically, it could also be due to a "lag effect" where digital investments have not yet yielded green outcomes. A more in-depth discussion or analysis using lag structures might be beneficial.

The Policy Implications are Somewhat Generic and Could be More Targeted

The policy recommendations are broad. It is suggested to make them more specific based on the empirical results. For example, propose green innovation subsidies for low-growth firms and stricter ESG disclosure requirements for high-growth firms.

Figure and Table Numbering is Inconsistent, with Citation Errors

The manuscript contains multiple instances of "Error! Reference source not found." throughout the text. A thorough proofread to correct all figure/table cross-references is necessary.

The Language is Occasionally Redundant and Could Benefit from Polishing

Some sections, particularly in the theory-building part, contain repetitive phrasing. Streamlining the language would improve readability and conciseness.

Reviewer #2: Reviewer Recommendation: Major Revision

The manuscript investigates the relationship between digital transformation and ESG performance in Chinese energy-intensive enterprises, exploring the mediating role of green innovation and the moderating effect of growth potential. While the research context (China's "dual carbon" goals) is highly relevant and the inclusion of growth potential as a negative moderator provides an interesting theoretical angle, the manuscript currently suffers from severe methodological flaws and a significant structural imbalance. To meet PLOS ONE’s rigorous criteria for statistical robustness and methodological validity, substantial revisions are required.

Below are the major and minor concerns that need to be addressed:

Majors

- The authors measure the mediating variable, "Green Innovation," as the natural logarithm of the number of green invention patent applications plus one. The descriptive statistics reveal that the median is 0, indicating that a significant portion of the sample has zero patent applications. Applying standard Ordinary Least Squares (OLS) regressions to count data with a heavy right skew and zero-inflation violates fundamental statistical assumptions (normality and homoscedasticity of residuals), leading to biased and inconsistent estimates.

- Need to address � The authors must re-estimate the models predicting green innovation using models appropriate for count or censored data, such as a Poisson regression, Negative Binomial regression, or a Tobit model.

The study uses panel data and claims a "direct impact" of digital transformation on ESG performance. To mitigate reverse causality, the independent variable is lagged by one period. In corporate finance and management research, a simple one-year lag is insufficient to resolve endogeneity concerns. Firms with stronger ESG performance often have better access to financing, allowing them to invest more heavily in digital transformation (simultaneity/reverse causality). Furthermore, the risk of omitted variable bias remains high.

- Need to address � The authors must implement a more robust identification strategy to address endogeneity. Acceptable approaches include Instrumental Variables (IV/2SLS), Generalized Method of Moments (GMM), or Propensity Score Matching with Difference-in-Differences (PSM-DID). Without these robustness checks, the paper can only claim correlations, not causal impacts.

The manuscript relies on the Baron and Kenny (1986) causal steps approach and the Sobel test to establish mediation. The Baron and Kenny method is widely considered outdated in modern econometrics and psychology, as it suffers from low statistical power. Similarly, the Sobel test assumes a normal distribution for the indirect effect, which is rarely the case.

- Need to address � The authors should use a Bootstrapping approach (e.g., using the PROCESS macro or Structural Equation Modeling) to test the significance of the indirect effect for the entire mediation model, replacing the Baron and Kenny framework entirely.

A disproportionate amount of the manuscript is dedicated to a CiteSpace bibliometric analysis simply to justify the choice of variables and identify the research gap. This creates a structural imbalance, making the manuscript read like two separate, disjointed papers. In an empirical financial/management paper, the research gap should be established through a concise, well-written literature review, not extensive keyword network mappings.

- Need to address � The bibliometric section should be drastically condensed (to 2-3 pages maximum) or moved entirely to the Supplementary Materials/Appendix. The core focus of the manuscript must be the econometric model, its robustness, and the economic interpretation of the findings.

Minors

- The authors state that the raw data were obtained from third-party databases (CSMAR and CNRDS). While providing links to these databases is helpful, PLOS ONE has a strict open-data policy.

- Need to address � To ensure perfect replicability without violating third-party copyright of the raw databases, the authors should upload their final, compiled, and cleaned dataset (e.g., in .csv or .dta format) along with the replication code (Stata do-files or R scripts) as Supporting Information.

- Several figures generated via CiteSpace suffer from poor resolution, overlapping text, and a confusing mix of English and Chinese characters.

- Need to address � All figures must be redesigned or exported at a higher resolution (minimum 300 dpi) to ensure all node labels are legible and clearly translated into English, in accordance with the journal's formatting guidelines.

- The manuscript occasionally uses hyperbolic language (e.g., "unprecedented," "exponential growth," "profoundly reflects").

- Need to address � Please review the manuscript to ensure a more objective, neutral, and scientifically rigorous tone throughout the text. Let the data and the statistical significance speak for the magnitude of the findings.

**********

what does this mean?). If published, this will include your full peer review and any attached files.

If you choose “no”, your identity will remain anonymous but your review may still be made public.

**Do you want your identity to be public for this peer review?**  For information about this choice, including consent withdrawal, please see our Privacy Policy

Reviewer #1: No

Reviewer #2: No

**********

[NOTE: If reviewer comments were submitted as an attachment file, they will be attached to this email and accessible via the submission site. Please log into your account, locate the manuscript record, and check for the action link "View Attachments". If this link does not appear, there are no attachment files.]

To ensure your figures meet our technical requirements, please review our figure guidelines: https://journals.plos.org/plosone/s/figures

You may also use PLOS’s free figure tool, NAAS, to help you prepare publication quality figures: https://journals.plos.org/plosone/s/figures#loc-tools-for-figure-preparation.

NAAS will assess whether your figures meet our technical requirements by comparing each figure against our figure specifications.

Attachment Submitted filename: Review Comments.docx

Attachment Submitted filename: Notes.docx

# Author comment: Author response to Decision Letter 1

**DOI:** 10.1371/journal.pone.0343345.r002

27 Jul 2026

I have carefully addressed all comments raised by the Editor and reviewers. A detailed point-by-point response is provided in the uploaded Response to Reviewers file. The manuscript has been substantially revised according to the suggestions, including revisions to the theoretical framework, empirical methodology, mechanism analysis, and presentation of findings.

Attachment Submitted filename: Response to Reviewers.docx

# Peer review: Decision Letter 1

**DOI:** 10.1371/journal.pone.0343345.r003

**Authors:** Juan Trinidad-Segovia

27 Aug 2026

Beyond Digital Investment: How Value Embedding Modes Shape ESG Performance in Energy-Intensive Firms

PONE-D-26-05685R1

Dear Dr. Weiran,

We’re pleased to inform you that your manuscript has been judged scientifically suitable for publication and will be formally accepted for publication once it meets all outstanding technical requirements.

Within one week, you’ll receive an e-mail detailing the required amendments. When these have been addressed, you’ll receive a formal acceptance letter and your manuscript will be scheduled for publication.

An invoice will be generated when your article is formally accepted. Please note, if your institution has a publishing partnership with PLOS and your article meets the relevant criteria, all or part of your publication costs will be covered. Please make sure your user information is up-to-date by logging into Editorial Manager at Editorial Manager® and clicking the ‘Update My Information' link at the top of the page. For questions related to billing, please contact billing support.

If your institution or institutions have a press office, please notify them about your upcoming paper to help maximize its impact. If they’ll be preparing press materials, please inform our press team as soon as possible -- no later than 48 hours after receiving the formal acceptance. Your manuscript will remain under strict press embargo until 2 pm Eastern Time on the date of publication. For more information, please contact onepress@plos.org.

Kind regards,

Juan E. Trinidad-Segovia, PhD

Section Editor

PLOS One

Additional Editor Comments (optional):

Reviewers' comments:

Reviewer's Responses to Questions

**Comments to the Author**

Reviewer #1: All comments have been addressed

Reviewer #3: All comments have been addressed

**********

2. Is the manuscript technically sound, and do the data support the conclusions?

Reviewer #1: Yes

Reviewer #3: Yes

**********

3. Has the statistical analysis been performed appropriately and rigorously?

Reviewer #1: Yes

Reviewer #3: Yes

**********

4. Have the authors made all data underlying the findings in their manuscript fully available?

The PLOS Data policy

Reviewer #1: No

Reviewer #3: Yes

**********

5. Is the manuscript presented in an intelligible fashion and written in standard English?

Reviewer #1: Yes

Reviewer #3: Yes

**********

Reviewer #1: In this round of revision, the author has provided comprehensive and thoughtful responses to all reviewer comments. It is particularly commendable that the author did not rigidly adhere to the original "green innovation mediation—growth moderation" model. Instead, based on the empirical results revealed by more rigorous econometric methods, the author proactively reconstructed the theoretical framework, upgrading the core explanatory logic from the "strength of a single mediator" to a comparison of the stability between "asset-embedded" and "information-embedded" pathways. This approach of driving theoretical refinement through empirical discovery demonstrates a high degree of scholarly rigor.

Prior to final acceptance, I recommend that the author address the following minor details:

Check the formatting of Table 5 and Table 6 to ensure no residual editing marks (e.g., "Formatted" tags) remain.

Review the reference list to confirm that all cited works appear in the main text and that all in-text citations have corresponding entries in the reference list.

Consider capitalizing core theoretical terms consistently (e.g., Resource-Based View, Dynamic Capabilities Theory, Stakeholder Theory) to align with standard academic conventions.

Regarding data availability: The author's statement that raw data are sourced from third-party commercial databases (CSMAR and CNRDS) and cannot be publicly redistributed due to licensing restrictions is reasonable and complies with PLOS ONE's exceptions for third-party data restrictions. However, I suggest that the author further clarify that the replication code (e.g., Stata do-files or R scripts) can be made available separately, enabling researchers with legitimate database access to reproduce the analysis. This would enhance the transparency and reproducibility of the study.

Reviewer #3: (No Response)

**********

what does this mean?). If published, this will include your full peer review and any attached files.

If you choose “no”, your identity will remain anonymous but your review may still be made public.

**Do you want your identity to be public for this peer review?**  For information about this choice, including consent withdrawal, please see our Privacy Policy

Reviewer #1: No

Reviewer #3: No

**********

# Editor report: Acceptance letter

**DOI:** 10.1371/journal.pone.0343345.r004

**Authors:** Juan Trinidad-Segovia

PONE-D-26-05685R1

PLOS One

Dear Dr. Sun,

I'm pleased to inform you that your manuscript has been deemed suitable for publication in PLOS One. Congratulations! Your manuscript is now being handed over to our production team.

At this stage, our production department will prepare your paper for publication. This includes ensuring the following:

* All references, tables, and figures are properly cited

* All relevant supporting information is included in the manuscript submission,

* There are no issues that prevent the paper from being properly typeset

You will receive further instructions from the production team, including instructions on how to review your proof when it is ready. Please keep in mind that we are working through a large volume of accepted articles, so please give us a few days to review your paper and let you know the next and final steps.

Lastly, if your institution or institutions have a press office, please let them know about your upcoming paper now to help maximize its impact. If they'll be preparing press materials, please inform our press team within the next 48 hours. Your manuscript will remain under strict press embargo until 2 pm Eastern Time on the date of publication. For more information, please contact onepress@plos.org.

You will receive an invoice from PLOS for your publication fee after your manuscript has reached the completed accept phase. If you receive an email requesting payment before acceptance or for any other service, this may be a phishing scheme. Learn how to identify phishing emails and protect your accounts at https://explore.plos.org/phishing.

If we can help with anything else, please email us at customercare@plos.org.

Thank you for submitting your work to PLOS One and supporting open access.

Kind regards,

PLOS One Editorial Office Staff

on behalf of

Dr. Juan E. Trinidad-Segovia

Section Editor

PLOS One
